# Early effects of LPS-induced neuroinflammation on the rat hippocampal glycolytic pathway

**DOI:** 10.1186/s12974-022-02612-w

**Published:** 2022-10-11

**Authors:** Adriana Fernanda K. Vizuete, Fernanda Fróes, Marina Seady, Caroline Zanotto, Larissa Daniele Bobermin, Ana Cristina Roginski, Moacir Wajner, André Quincozes-Santos, Carlos Alberto Gonçalves

**Affiliations:** 1 Laboratory of Calcium-Binding Proteins in the CNS, Department of Biochemistry, Institute of Basic Health Sciences, Universidade Federal Do Rio Grande Do Sul (UFRGS), Ramiro Barcelos, 2600-Anexo, Porto Alegre, RS Zip Code: 90035-003 Brazil; 2 Pos Graduate Program in Biochemistry, Institute of Basic Health Sciences, UFRGS, Porto Alegre, RS Brazil; 3 Department of Biochemistry, Institute of Basic Health Sciences, UFRGS, Porto Alegre, RS Brazil; 4 Medical Genetics Service, Hospital de Clínicas de Porto Alegre, Porto Alegre, RS Brazil

**Keywords:** Neuroinflammation, Glycolysis, Glucose uptake, PFK1, S100B, IL-1β

## Abstract

Neuroinflammation is a common feature during the development of neurological disorders and neurodegenerative diseases, where glial cells, such as microglia and astrocytes, play key roles in the activation and maintenance of inflammatory responses in the central nervous system. Neuroinflammation is now known to involve a neurometabolic shift, in addition to an increase in energy consumption. We used two approaches (in vivo and ex vivo) to evaluate the effects of lipopolysaccharide (LPS)-induced neuroinflammation on neurometabolic reprogramming, and on the modulation of the glycolytic pathway during the neuroinflammatory response. For this, we investigated inflammatory cytokines and receptors in the rat hippocampus, as well as markers of glial reactivity. Mitochondrial respirometry and the glycolytic pathway were evaluated by multiple parameters, including enzymatic activity, gene expression and regulation by protein kinases. Metabolic (e.g., metformin, 3PO, oxamic acid, fluorocitrate) and inflammatory (e.g., minocycline, MCC950, arundic acid) inhibitors were used in ex vivo hippocampal slices. The induction of early inflammatory changes by LPS (both in vivo and ex vivo*)* enhanced glycolytic parameters, such as glucose uptake, PFK1 activity and lactate release. This increased glucose consumption was independent of the energy expenditure for glutamate uptake, which was in fact diverted for the maintenance of the immune response. Accordingly, inhibitors of the glycolytic pathway and Krebs cycle reverted neuroinflammation (reducing IL-1β and S100B) and the changes in glycolytic parameters induced by LPS in acute hippocampal slices. Moreover, the inhibition of S100B, a protein predominantly synthesized and secreted by astrocytes, inhibition of microglia activation and abrogation of NLRP3 inflammasome assembly confirmed the role of neuroinflammation in the upregulation of glycolysis in the hippocampus. Our data indicate a neurometabolic glycolytic shift, induced by inflammatory activation, as well as a central and integrative role of astrocytes, and suggest that interference in the control of neurometabolism may be a promising strategy for downregulating neuroinflammation and consequently for diminishing negative neurological outcomes.

**Supplementary Information:**

The online version contains supplementary material available at 10.1186/s12974-022-02612-w.

## Highlights

- LPS-induced neuroinflammation promotes metabolic reprogramming.
- Neuroinflammation increased glycolytic flow and lactate release.
- Inhibitors of different glycolytic reactions prevented the inflammatory response.
- S100B changes suggest a role for astrocytes in this metabolic shift.
- The glycolytic pathway should be target for new anti-inflammatory strategies.

**Graphical Abstract:**

**Supplementary Information:**

The online version contains supplementary material available at 10.1186/s12974-022-02612-w.

## Graphical abstract

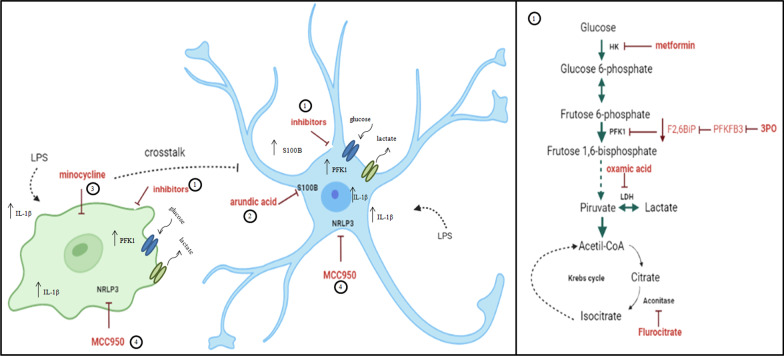

## Introduction

Neuroinflammation is an inflammatory event that occurs in the nervous tissue and involves intense communication between the immune system and the central nervous system (CNS). This event constitutes complex cellular and molecular responses to pathogens and/or the release of cellular debris and intracellular molecules (S100B, HMGB1, etc.) due to exposure to a harmful environment, such as hypoxia, low energy support, neurotoxicity, or oxidative stress, etc. Acute and chronic inflammatory signaling in CNS is associated with the development of several neurodegenerative diseases and neurological disorders [1–4]. Several hallmarks have been described as common features of neurodegenerative disease and neuroinflammation [5]. However, the role of this pathway as a cause or as a consequence of neuronal dysfunction remains unclear.

Glial cells, such as microglia and astrocytes, are considered innate immune cells in the CNS. These cells synthesize and secrete several inflammatory mediators in order to maintain brain tissue homeostasis during physiological and pathophysiological responses [6, 7]. In fact, neurotoxic insults (drugs, cellular debris, pathogens, etc.) promote morphological, metabolic and molecular changes in glial cells, denominated reactive gliosis. Reactive microglia and astrocytes release pro-inflammatory cytokines, such as tumor necrosis factor alpha (TNF-α) and interleukin-1 beta (IL-1β), which leads to glutamate neurotoxicity and neuronal death, events related to neurodegenerative diseases [8–10].

The activation and maintenance of the immune response in glial cells is dependent on a neurometabolic shift and an increase in energy consumption, mediated by the glycolysis pathway [11, 12]. In fact, in the neuroinflammatory scenario, reactive astrocytes become more glycolytic than they already are [13], and elevations in gene expression and in the activity of 6-phosphofructose-2-kinase/fructose-2,6-bisphosphatase isoform 3 (PFKFB3) occurs, promoting increased synthesis of fructose-2,6-bisphosphate (fructose 2,6 biP), an allosteric activator of the enzyme, 6-phosphofructo-1-kinase (PFK1), and consequent upregulation of the glycolytic pathway [14]. Moreover, in this context, reactive microglia also upregulate the non-oxidative glycolysis pathway [15]. Recent studies have demonstrated increased glycolytic pathway activity in association with the responses of peripheral immune cells, an event similar to the Warburg phenomenon observed in tumor cells [16, 17]. It is believed that this metabolic change also occurs in glial cells, in response to the neuroinflammatory process.

The lipopolysaccharide (LPS)-induced inflammation model is widely used, including in the CNS [18]. In fact, all neural cells express toll-like receptor 4 (TLR 4), the main receptor for LPS, whose activation results in receptor dimerization and recruitment of intracellular adapter proteins, which ultimately leads to activation of the NF-kB signaling pathway and cytokine production [19, 20].

Considering that changes in glycolytic energy metabolism in the CNS are related to neuroinflammation signaling and neurodegenerative disease development, the regulation of key glycolytic enzymes may represent a target for the study of the energy consumption in the inflammatory and metabolism energetic response. On the other hand, inhibition of neuroinflammation could affect glycolytic metabolism and its reactive response. Thus, the purpose of this study was to evaluate the effect of LPS-induced neuroinflammation on neurometabolism in vivo and ex vivo, and the modulation of the glycolytic pathway during the neuroinflammatory response.

## Methods

### Materials

Lipopolysaccharide from *Escherichia coli* (LPS, 055:B5), TRI Reagent, minocycline, 3-(3-pyridinyl)-1-(4-pyridinyl)-2-propen-1-one (3PO), metformin, oxamic acid, MCC950, fluorocitrate, PFK1 activity assay, anti-S100B (SH-B1), 4-(2-hydroxyethyl) piperazine-l-ethanesulfonic acid (HEPES), *o*-phenylenediamine (OPD), and HRP- conjugated anti-goat IgG and anti-p38 MAPK were purchased from Sigma (Saint Louis, MO, USA). Arundic acid was purchased from TOCRIS (Bristol, United Kingdom). Standard GFAP was from Calbiochem (San Diego, CA, USA). Polyclonal anti-S100B and polyclonal anti-GFAP were purchased from DAKO (Carpinteria, CA, U.S.A.). The lactate and lactate dehydrogenase (LDH) assays were purchased from BioClin, Brazil. Polyclonal anti-EAAT2 (GLT1) and anti-EAAT1 (GLAST) were purchased from Abcam (Cambridge, MA, USA) and anti-GLUT1, anti-COX2, anti-TLR4 and anti-RAGE were purchased from Santa Cruz Biotechnology (Inc., Dallas, Texas, USA). Monoclonal anti-Iba1 was purchased from Merck/Millipore (Darmstadt, Germany). Anti-phospho p38 MAPK, anti-Akt and phospho Akt (Ser473) were purchased from Cell Signaling Technology (Danvers, Massachusetts, U.S.A.). Anti-HRP conjugated actin was purchased from Proteintech (Rosemento, IL, USA). Finally, HRP-conjugated anti-rabbit IgG and anti-mouse IgG were purchased from GEHealthcare (Little Chalfont, United Kingdom).

### Animals

Fifty-four male *Wistar* rats were obtained from our breeding colony (Department of Biochemistry, UFRGS), at postnatal day 30, and maintained under controlled light and environmental conditions (12 h light/12 h dark cycle at a constant temperature of 22 ± 1 °C). Procedures were in accordance with the National Institute of Health Guide for the Care and Use of Laboratory Animals (NIH Publications No. 80–23) and followed the regulations of the local animal house authorities and the Committee of Animal Use of UFRGS (project number 38546).

### First study—in vivo neuroinflammation model

Eighteen animals were subjected to the in vivo LPS neuroinflammation induction model, according to a previous study [21]. Briefly, rats were treated intraperitoneally with LPS (1 mg/kg, *i.p*.) or saline (0.9% NaCl, *i.p.*), and divided into three groups: (1) Sham (vehicle), (2) LPS 6 h (euthanized at 6 h after LPS administration) and (3) LPS 24 h (euthanized at 24 h after LPS administration).

#### Brain tissue, serum and CSF samples

Rats were anaesthetized by *i.p.* injection of ketamine (75 mg/kg) and xylazine (10 mg/kg). For ventricular access, the anesthetized rats were placed in a stereotaxic apparatus and cerebrospinal fluid (CSF) was obtained by carefully puncturing the cisterna magna with an insulin syringe. A maximum volume of 30 μl was collected over a 3-min period to minimize the risk of brain stem damage. Blood was collected by cardiac puncture and serum was obtained by centrifuging at 1000×*g* for 10 min (Eppendorf 5402, Hamburg, Germany), before storing at − 80 °C. Hippocampi were dissected and transverse slices of 0.3 mm were obtained using a McIlwain Tissue Chopper, as described above. Samples were stored at − 80 °C until biochemical and immunological assays.

### Second study—ex vivo neuroinflammation model

#### Preparation and incubation of hippocampal slices

Thirty-six naïve animals were used for preparing ex vivo acute hippocampal slices. Briefly, animals were euthanized by decapitation; their brains were removed and placed in cold saline medium of the following composition (in mM): 120 NaCl; 2 KCl; 1 CaCl_2_; 1 MgSO_4_; 25 HEPES; 1 KH_2_PO_4_ and 10 glucose, adjusted to pH 7.4. The hippocampi were dissected and transverse slices of 0.3 mm were obtained using a McIlwain Tissue Chopper. Slices were then transferred immediately to 24-well culture plates, each well containing 0.3 mL of physiological medium and only one slice. The medium was replaced every 15 min with fresh saline medium at room temperature. Following a 120-min equilibration period, the medium was removed and replaced with basal or specific treatments for 60 min at 30 °C on a warm plate [22, 23]. Slices were incubated with the following treatments: normal saline, LPS (10 μg/mL), metformin (Met, 500 μM), 3-(3-pyridinyl)-1-(4-pyridinyl)-2-propen-1-one (3PO, 20 μM), which inhibits the PFK-2 (6-phosphofructo-2-kinase) activity of PFKFB3, oxamic acid (a non-competitive inhibitor of LDH; OA, 10 μM), fluorocitrate (inhibits aconitase; FLC, 10 μM), arundic acid (putative inhibitor of astrocyte S100B synthesis and secretion; AA, 100 μM), the microglia cell inhibitor, minocycline (Mino,10 μM), and the NLRP3 inhibitor, MCC950 (MCC, 10 μM). All agents were used at doses based on previous studies [21, 24, 25] and based on the glucose uptake response curve (Additional file 1: Figure S1).

### Cytokine measurement

Hippocampal slices were homogenized in phosphate buffer saline (PBS) containing (in mM) 50 NaCl, 18 Na_2_HPO_4_, 83 NaH_2_PO_4_.H_2_O, pH 7.4, with 1 mM EGTA and 1 mM phenylmethyl-sulphonyl fluoride (PMSF), followed by centrifugation at 1000×*g* for 5 min at 4 °C. Cytokines were measured in supernatants and extracellular medium samples using commercial rat TNF-α and IL-1β ELISAs, according to the manufacturer’s instructions (eBioscience, San Diego, USA). Data are expressed in pg/mg protein (tissue samples) or pg/mL (serum and extracellular medium).

### S100B measurement

Slices were homogenized in PBS with 1 mM EGTA and 1 mM PMSF. The S100B contents in the CSF, serum, brain tissue and extracellular medium were measured by ELISA, as described previously [26]. Briefly, 50 µL of sample plus 50 µL of Tris buffer were incubated for 2 h on a microtiter plate that was previously coated with monoclonal anti-S100B SH-B1. Polyclonal anti-S100 was incubated for 30 min and peroxidase-conjugated anti-rabbit antibody was then added for a further 30 min. The color reaction with OPD was measured at 492 nm. The standard S100B curve ranged from 0.02 to 10 ng/mL. Data are expressed in ng/mg protein (tissue samples) or ng/mL (CSF, serum and extracellular medium).

### GFAP measurement

GFAP content in the hippocampus tissue was measured by ELISA, as described previously [27]. The ELISA for GFAP was carried out by coating wells of 96-well plates with 100 μL samples containing 70 μg of protein, overnight at 4 °C. Wells were incubated with a rabbit polyclonal anti-GFAP antibody (Dako, Carpinteria, CA, U.S.A.) for 2 h, followed by incubation with a secondary antibody conjugated with peroxidase for 1 h, at room temperature. The color reaction with OPD was measured at 492 nm. The standard GFAP curve ranged from 0.1 to 10 ng/mL. Data are expressed in ng/mg protein.

### RNA extraction and quantitative RT-PCR

Total RNA was isolated from hippocampal tissue using TRI reagent. The concentration and purity of the RNA were determined spectrophotometrically at a ratio of 260:280. Subsequently, 1 μg of total RNA was reverse transcribed using Applied Biosystems High Capacity complementary DNA (cDNA) with a Reverse Transcription Kit in a 20 μL reaction, according to the manufacturer’s instructions. The messenger RNAs (mRNAs) encoding TLR2 (#Rn02133647_s1), TLR4 (#Rn00569848_m1), RAGE (#Rn01525753_g1), NLRP3 (#Rn04244620_m1), IL-1β (#Rn00580432_m1), IL1R1 (#Rn00565482_m1), TNF-α (#Rn99999017_m1), TNFR1 (#Rn01492348_m1), G6PD (#Rn01529640_g1), and AMPK (#Rn00576935_m1) were quantified using the TaqMan real-time RT-PCR system, employing inventory primers and probes purchased from Applied Biosystems. Quantitative RT-PCR was performed using the Applied Biosystems StepOne System. Target mRNA levels were normalized to β-actin (#Rn00667869_m1) levels. The results were analyzed employing the 2^−ΔΔCt^ method [28] and expressed relative to the levels of the control group.

### Western blotting

Nitrocellulose membranes were blocked overnight at 4 °C with 2% bovine serum albumin (BSA) in Tris-buffered saline (TBS), in mM; 10 Tris, 150 NaCl, pH 7.5 and 0.05% Tween 20®, and then incubated overnight at 4 °C in blocking solution containing the following antibodies, anti-COX2, anti-TLR4, anti-RAGE, anti-GLUT1, anti-EAAT2 (GLT1), anti-EAAT1 (GLAST), anti-Iba1, anti-HRP conjugated actin (1:20,000), antip-38 MAPK, anti-phospho p38MAPK (Thr18/Tyr182), anti-Akt and anti-phospho Akt (Ser473) (Additional file 7: Table S1). Subsequently, membranes were incubated for 1 h at room temperature in solution containing horseradish peroxidase (HRP)-conjugated anti-rabbit IgG, HRP-conjugated anti-mouse IgG or HRP-conjugated anti-goat. A chemiluminescence signal was detected by a luminol substrate reaction (ECL Western Blotting System, GE Healthcare®). Immunoblots were quantified by membrane scanning in an Image4000 (GE Healthcare®), optical densities of proteins studied were determined by ImageJ software (Packard Instrument Company) and the protein/actin ratio was calculated.

### Determination of mitochondrial respiratory parameters by oxygen consumption

Oxygen consumption was measured in hippocampus crude homogenates (2 mg/mL tissue) using a substrate-uncoupler inhibitor titration (SUIT) protocol [29]. NADH-linked substrates (5 mM pyruvate, 0.5 mM malate and 10 mM glutamate) were first added to the chamber, followed by 500 μM ADP (state 3 respiration PMG), and 10 mM of succinate (FADH_2_-linked substrate) were then added (state 3 respiration PMG + S). To evaluate state 4 respiration, oligomycin (1 μg mL^−1^) was added to the chamber. Next, 1.5 μM CCCP (three pulses of 0.5 μM) was titrated to determine electron transfer system (ETS) capacity (non-coupled respiration PMG + S). Rotenone (1 μM; complex I inhibitor) was used to obtain the non-coupled respiration stimulated by succinate (Non-coupled respiration S). Finally, antimycin A was added to the chamber for the determination of ROX. All parameters were corrected by ROX.

The real-time oxygen fluxes were calculated using DatLab7 (Oroboros Instruments) and expressed as pmol O_2_ flux·s^−1^·mg protein^−1^.

### Glutamate uptake

The glutamate uptake assay was performed as previously described [30], with some modifications. Hippocampal slices were obtained and transferred to 24-well culture plates with Hank’s balanced salt solution (HBSS) containing (in mM) 137 NaCl, 5.36 KCl, 1.26 CaCl_2_, 0.41 MgSO_4_, 0.49 MgCl_2_, 0.63 Na_2_HPO_4_·7H_2_O, 0.44 KH_2_PO_4_, 4.17 NaHCO_3_, and 5.6 glucose, adjusted to pH 7.2, at 37 °C. The assay was initiated by the addition of 0.1 mM L-glutamate and 0.33 μCi/mL L-[2,3-^3^H] glutamate. The incubation was stopped after 5 min by removing the medium and rinsing the slices twice with ice-cold HBSS. Hippocampus tissue was then lysed in a 0.5 M NaOH solution. Sodium-independent uptake was determined using N-methyl-D-glucamine instead of NaCl. Sodium-dependent glutamate uptake was obtained by subtracting the nonspecific uptake from the total uptake to obtain the specific uptake. Radioactivity was measured in a scintillation counter. Results are expressed as nmol/mg protein/min.

### Glucose uptake

The glucose uptake assay was performed with hippocampal slices, as previously described [31]. Briefly, slices were incubated at 35 °C in HBSS. The assay was initiated by the addition of 0.1 µCi/ well D-[3-^3^H] glucose. The incubation was stopped after 30 min by removing the medium and rinsing the slices twice with ice-cold HBSS. Hippocampus tissue was then lysed in a 0.5 M NaOH solution. Radioactivity was measured using a scintillation counter. Glucose uptake was calculated by subtracting the non-specific uptake, obtained by the glucose transport inhibitor, cytochalasin B (25 µM), from the total uptake. This assay demonstrates glucose uptake as an indirect measurement of intracellular tritiated-glucose and derived metabolites. Radioactivity was measured in a scintillation counter. Results are expressed as nmol/mg protein/min.

### Glutathione content

Reduced glutathione (GSH) content was determined based on Allen et al. [32]. Briefly, slices were homogenized in sodium phosphate buffer (0.1 M, pH 8.0) and protein was precipitated with 1.7% meta-phosphoric acid. *O*-phthaldialdehyde (1 mg/ml methanol) (Sigma-Aldrich, St. Louis, MO. USA) was added to the supernatant at room temperature for 15 min. Fluorescence was measured using excitation and emission wavelengths of 350 and 420 nm, respectively. The standard calibration glutathione (Sigma) solution curve ranged from 0 to 500 μM. Glutathione results are expressed as nmol/mg protein.

### Reactive oxygen species (ROS) assay

Reactive oxygen species (ROS) were estimated by spectrofluorometry in the hippocampus using 2′7’- dichlorodihydrofluorescein diacetate (DCFH-DA), which is converted by ROS into highly fluorescent to 2′7’-dichlorofluorescein (DCF) (protocol modified from Lu et al. [33]). The resulting hippocampal homogenate was incubated with DCF-DA (5 µM) for 30 min at 37ºC in a water bath. Data are expressed in AU/mg protein.

### Protein measurement

Protein was measured by Lowry’s method, modified by Peterson, using bovine serum albumin as a standard [34].

### Statistical analysis

The experiments were performed at different time points of LPS neuroinflammation treatment (6 or 24 h). Acute hippocampal slices were analyzed in different periods and independently. Initially, data normality was evaluated using Shapiro–Wilk test and the results were considered parametric. All results were expressed as mean ± standard error mean (SEM). Multiple comparisons among groups were performed by one-way analysis of variance (ANOVA) followed by Tukey’s test and unpaired Student *t* test. The level of statistical significance was set at *P* < 0.05. All analyses were performed using the Prism 7.0 software (GraphPad).

## Results

In order to investigate the effect of neuroinflammation on energetic metabolism in the hippocampus structure, we used two neuroinflammatory models; firstly, an in vivo model, in which we evaluated neuroinflammation at 6 and 24 h after *i.p.* LPS injection. In a second model, ex vivo acute hippocampal slices were incubated with LPS for 1 h. We evaluated the activation of neuroinflammatory signaling and the effect on neurometabolism. Furthermore, we investigated the effects of glycolytic pathway and inflammatory modulation on neurometabolism and on the neuroinflammatory response.

### LPS induces neuroinflammation signaling in vivo

**Fig. 1:**
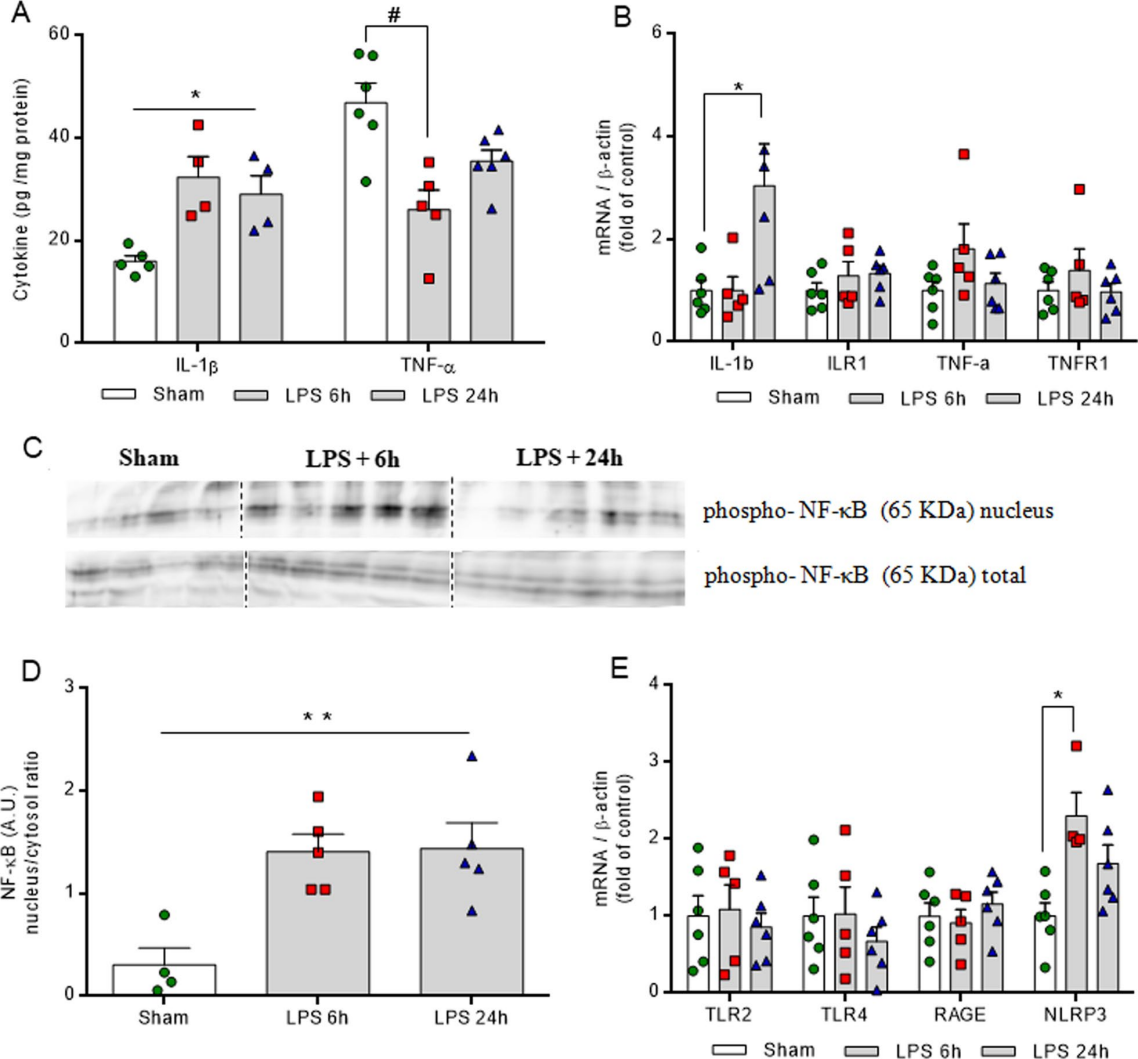
LPS activates neuroinflammatory pathways in the hippocampus. Pro-inflammatory immunocontent of cytokines (IL-1β and TNF-α) was measured by ELISA. Gene expressions of cytokines and receptors were evaluated by RT-PCR. Phospho-NFκB was evaluated in total homogenate and nucleus fraction by Western blot. LPS increases IL-1β at both time points, and at 6 h TNF-α was decreased (**A**). IL-1b mRNA increases only at 24 h after LPS injection, with no changes in its receptor (ILR1), and in TNF-α or its receptor, TNFR1 (B). Representative image of Western blot data (**C**). The translocation to the nucleus of phospho-NF-κB increases at both time points (**D**). No changes in mRNA of receptors (TLR2, TLR4, RAGE) involved in neuroinflammatory signaling. NLRP3 mRNA was increased only at 6 h (**E**). Values are expressed as means ± standard error. LPS 6 h (euthanized at 6 h after LPS administration), LPS 24 h (euthanized at 24 h after LPS administration). Data were analyzed by ANOVA, followed by the Tukey test, assuming *P* < 0.05. * means significant increase, when compared to sham group (* *P* < 0.05, ** *P* < 0.01), # means significant decrease, when compared to sham group

Firstly, in the in vivo model, LPS raised the hippocampal concentrations of the pro-inflammatory cytokine, IL-1β, at both time points (6 and 24 h after *i.p.* LPS injection) (Fig. 1A, *F*_(2,10)_ = 9.022,* P* = 0.0058). The levels of IL1b mRNA increased only after 24 h (Fig. 1B, *F*_(2,14)_ = 5.044, *P* = 0.0224), without changes in the expression of its receptor, ILR1. TNF-α cytokine levels decreased at 6 h after LPS administration (Fig. 1A, *F*_(2,14)_ = 9.592, *P* = 0.0024), without any change in the mRNA level of the cytokine and its receptor, TNFR1 (Fig. 1B). Furthermore, the translocation of the transcription factor, NF-κB, to the nucleus was observed at both time points (Fig. 1C and D *F*_(2,11)_ = 9.096, *P* = 0.0047) and mRNA encoding the NLRP3 inflammasome protein increased at the 6 h time point (Fig. 1E, *F*_(2,13)_ = 7.139, *P* = 0.0081). No changes in the levels of mRNA encoding receptors, such as TLR2, TLR4 and RAGE were observed at either of the time points.

### LPS induces astrocyte reactivity in vivo

**Fig. 2:**
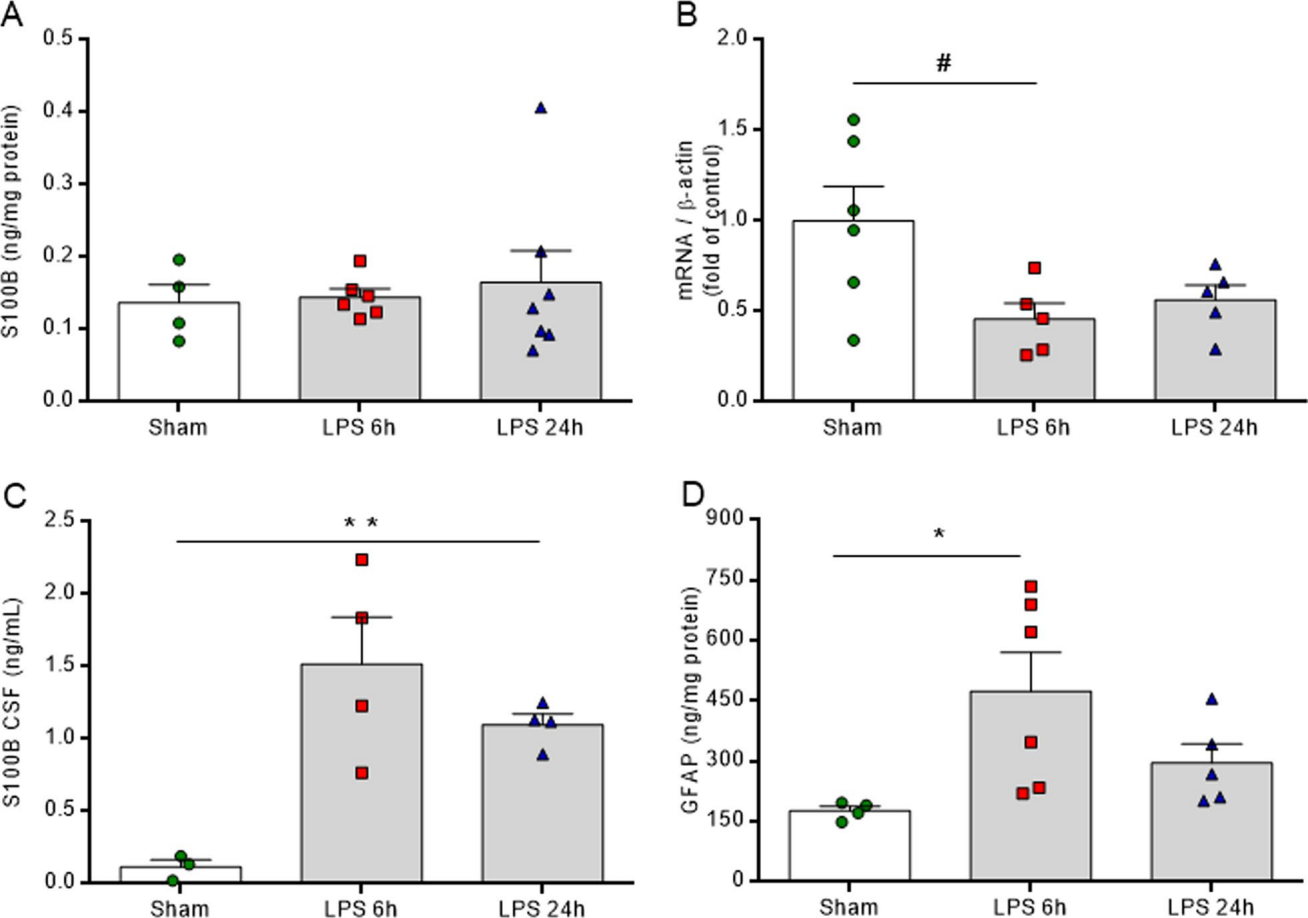
LPS induces astrocyte reactivity in the hippocampus. S100B and GFAP were measured by ELISA, and S100B gene expression was evaluated by RT-PCR. LPS reduced S100B gene expression at both times (**B**), without changes in protein content in the hippocampus (**A**) and elevation of S100B protein levels in the CSF (**C**). LPS raises GFAP immunocontent only at 6 h. LPS 6 h (euthanized at 6 h after LPS administration), LPS 24 h (euthanized at 24 h after LPS administration). Values are expressed as means ± standard error. Data were analyzed by ANOVA, followed by the Tukey test, assuming *P* < 0.05. * means significant increase, when compared to sham group (* *P* < 0.05, ** *P* < 0.01), # means significant decrease, when compared to sham group

LPS was also able to modulate astrocyte biomarkers, such as S100B and GFAP, in the hippocampus, as well as astrocyte metabolism. At both time points, reductions in S100B mRNA levels were observed (Fig. 2B, *F*_(2,13)_ = 4.486, *P* = 0.0330), without changes in protein immunocontent (Fig. 2A, *F*_(2,14)_ = 0.1835, *P* = 0.8343). LPS probably promoted a general effect on S100B secretion in the brain, raising the protein levels in the cerebrospinal fluid (CSF) (Fig. 2C, *F*_(2,8)_ = 10.250, *P* = 0.0062). Moreover, at 6 h, the GFAP immunocontent was increased in the hippocampal structure (Fig. 2D, *F*_(2,12)_ = 4.211, *P* = 0.0412). LPS administration (*i.p.*) also promoted peripheral inflammation and raised serum TNF-α and S100B protein levels at 6 h. Additionally, in general, LPS altered the peripheral energetic metabolism by reducing glycemia at both time points and by increasing the serum lactate level at 6 h (Additional file 2: Figure S2). The inflammatory and astrocyte reactivity scenario induced a dysfunction and an impairment in glutamate uptake at 6 h in the hippocampus, followed by a reduction in GSH content at both times, without changes in total reactive oxygen species (based on DCF assay) (Additional file 3: Figure S3).

### LPS induces glycolytic parameters in vivo

**Fig. 3:**
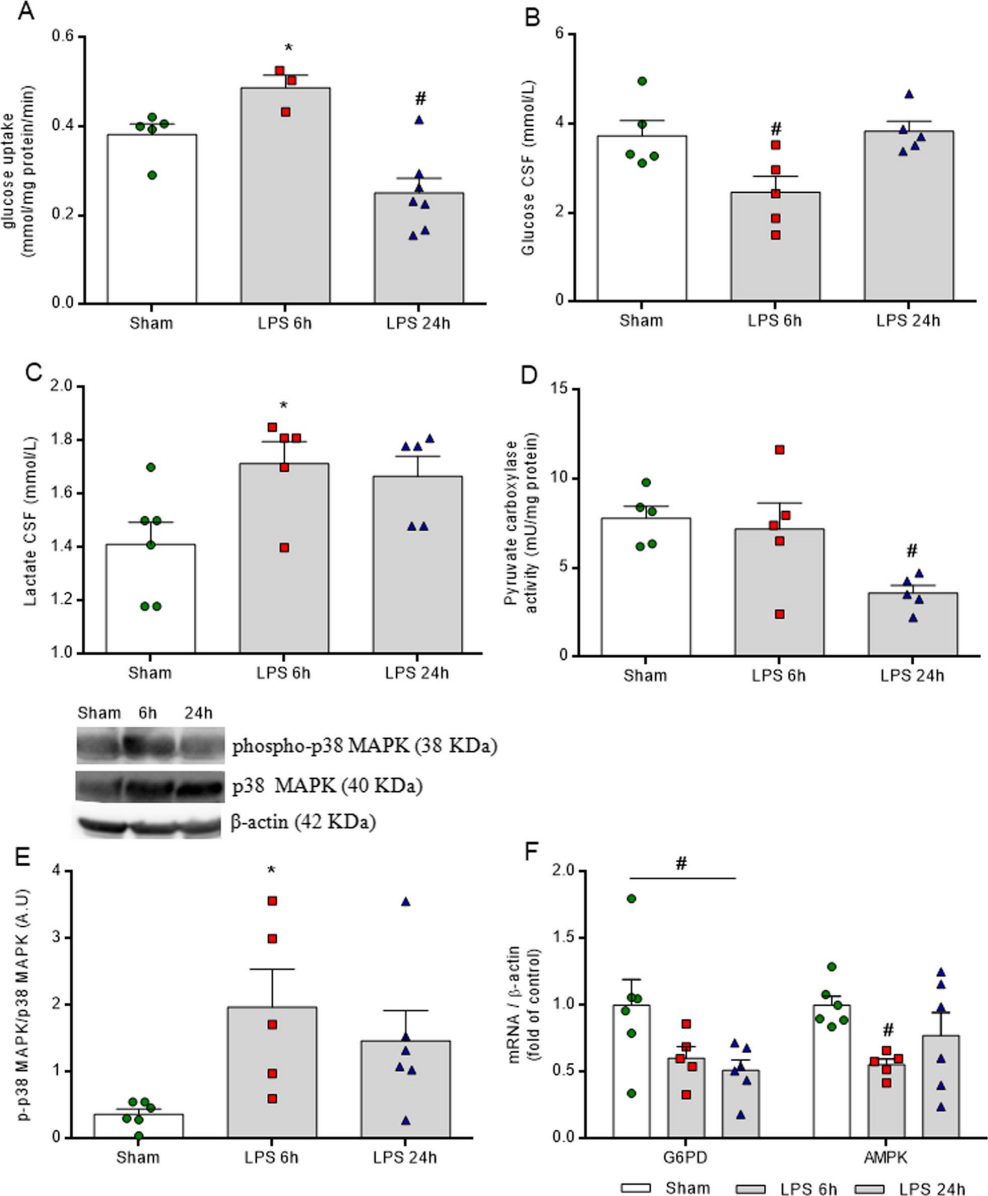
LPS induces glycolytic changes in the hippocampus. Glucose uptake was measured by radioactivity assay. Glucose and lactate serum levels were evaluated by spectrophotometric method. Pyruvate carboxylase activity was analyzed by kinetic assay. The phosphorylation of p38 MAPK was evaluated in total homogenates by Western blot and the gene expressions of G6PD and AMPK enzymes were evaluated by RT-PCR. LPS promotes a biphasic response in energy metabolism and, at 6 h, glucose uptake increases, in contrast to observations at 24 h, when glucose uptake is reduced (**A**). Energy metabolism intermediates were changed in the CSF only at 6 h after neuroinflammation. Glucose levels reduced (B), while lactate levels increased (**C**) in the CSF. Phosphorylation of p38 MAPK (**D**) was increased and the gene expression of G6PD decreased at both time points after LPS injection (**E**). AMPK expression was reduced only at 6 h (**E**). LPS 6 h (euthanized at 6 h after LPS administration), LPS 24 h (euthanized at 24 h after LPS administration). Values are expressed as means ± standard error. Data were analyzed by ANOVA, followed by the Tukey test, assuming *P* < 0.05. * means significant increase, when compared to sham group, # means significant decrease, when compared to sham group

The induction of neuroinflammation also affected neuroenergetic metabolism. At 6 h, glucose uptake increased (Fig. 3A, *F*_(2,12)_ = 12.560, *P* = 0.0011) and this energetic event correlated with low glucose levels (Fig. 3B, *F*_(2,12)_ = 5.854, *P* = 0.0168) and high lactate concentrations in the CSF (Fig. 3C, *F*_(2,13)_ = 4.207, *P* = 0.0390). At this time point, the changes in neurometabolism were followed by an increase in the phosphorylation of the p38 MAPK enzyme (Fig. 3E, *F*_(2,14)_ = 4.117, *P* = 0.0393) and reductions in mRNA for enzymes G6PD and AMPK (Fig. 3F, *F*_(2,14)_ = 3.793, *P* = 0.0483 and *F*_(2,14)_ = 3.612, *P* = 0.0500, respectively). In contrast, at 24 h, the glucose uptake decreased without affecting glucose and lactate CSF levels. At this time point, pyruvate carboxylase activity decreased (Fig. 3D, *F*_(2,10)_ = 3.954, *P* = 0.0500) and only the mRNA coding for G6PD decreased.

**Fig. 4:**
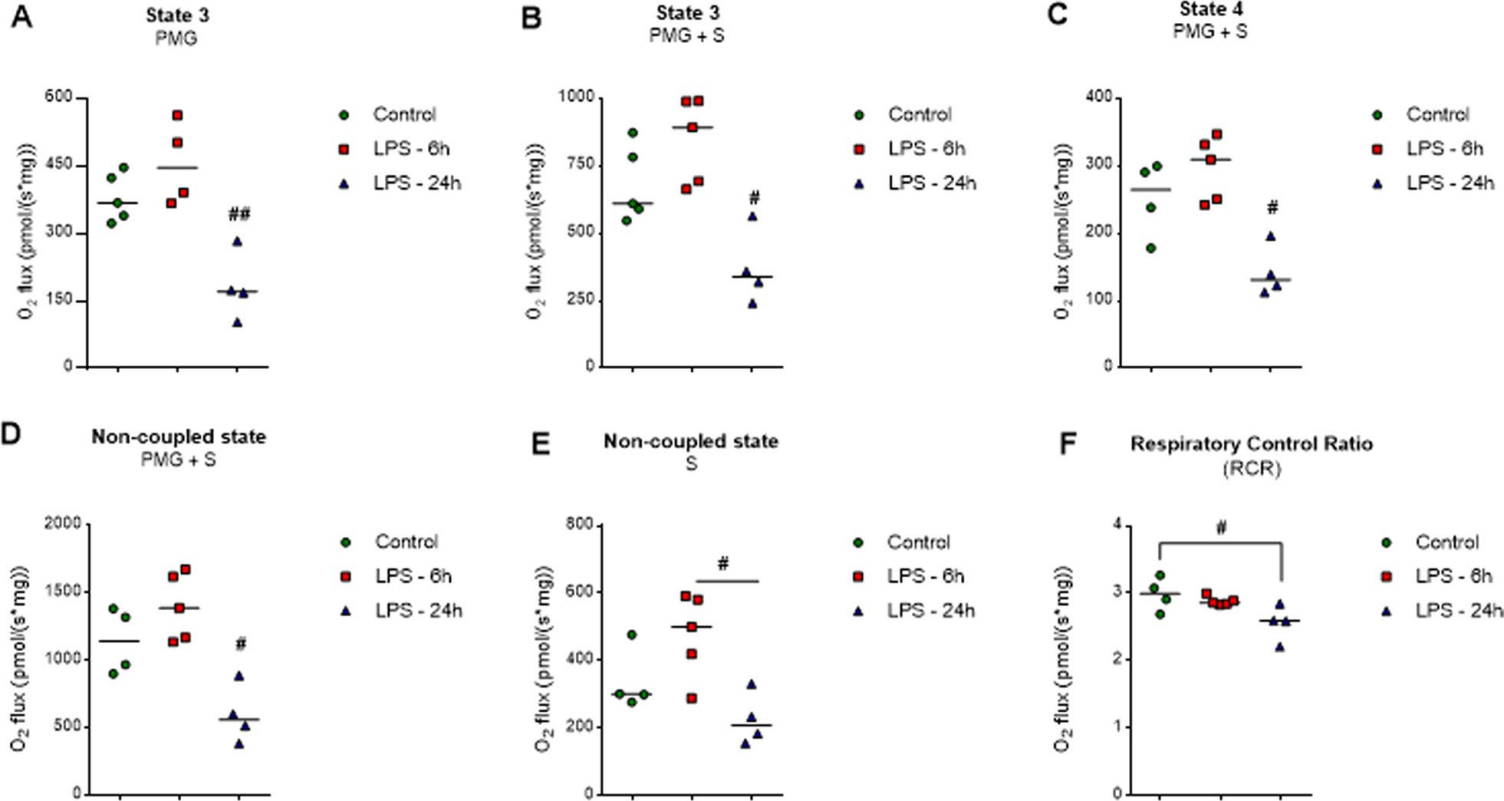
LPS induces mitochondrial impairment in the hippocampus. Oxygen consumption was measured in mitochondrial preparations from hippocampi. After 24 h, LPS reduced state 3 (ADP-stimulated) in the presence of metabolites PMC (**A**) and PMC + S (**B**) and state 4 (non-phosphorylating) in PMC + S (**C**). This time point decreases both uncoupled (CCCP-stimulated) (**D**, **E**) and the RCR (**F**). LPS 6 h (euthanized at 6 h after LPS administration), LPS 24 h (euthanized at 24 h after LPS administration). Values are expressed as means ± standard error. Data were analyzed by ANOVA, followed by the Tukey test, assuming *P* < 0.05. # means significant decrease, when compared to sham and LPS 6 h groups (# *P* < 0.05, ## *P* < 0.01)

The findings that mitochondrial activity and respirometry changes occurred in a time-dependent manner in the hippocampus of this model further supports a role for neuroinflammation in the modulation of neurometabolism. LPS reduced both the ADP-stimulated state and oligomycin-insensitive state of the mitochondria only at 24 h, with no alterations in most parameters at the time point of 6 h. The reduction of state 3 occurred with the metabolites, pyruvate/malate/glutamate (PMG) (Fig. 4A, *F*_(2,10)_ = 15.000, *P* = 0.0010) and PMG + succinate (PMG + S) (Fig. 4B, *F*_(2,11)_ = 11.860, *P* = 0.0229). The oligomycin-insensitive state 4 also decreased (Fig. 4C, *F*_(2,10)_ = 11.810, *P* = 0.0219), and we observed an impairment of the coupling of the respiratory chain with the synthesis of ATP (Fig. 4D, E, *F*_(2,10)_ = 12.940, *P* = 0.0214 and F_(2,10)_ = 6.576, *P* = 0.0122, respectively), as shown by the S3/S4 ratio (Fig. 4F, *F*_(2,10)_ = 4.867, *P* = 0.0345). As such, these findings lead us to postulate that this inflammation may promote a time-dependent metabolic shift. Furthermore, it is possible that 6 h of acute neuroinflammation induction was not enough time to modulate mitochondrial respiratory activity and that early inflammation was inducing the glycolytic pathway, since we observed elevations both in glucose uptake in the hippocampus and in lactate levels in the CSF, without changes in respirometry parameters.

### LPS induces neuroinflammation and astrocyte reactivity in acute hippocampal slices

**Fig. 5:**
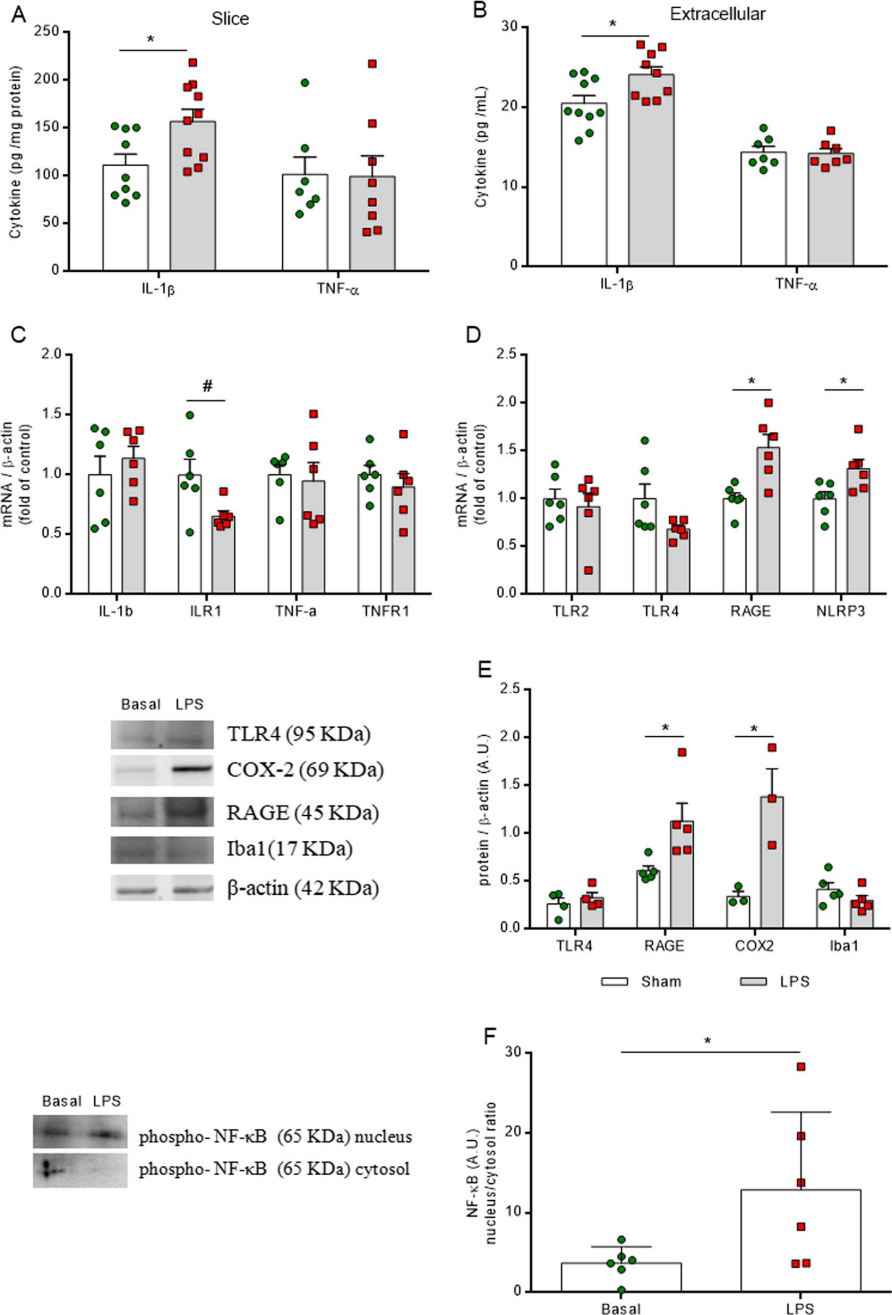
LPS promotes early neuroinflammation in acute hippocampal slices. Pro-inflammatory immunocontent of cytokines (IL-1β and TNF-α) was measured by ELISA in intracellular and extracellular medium. Gene expressions of cytokines and receptors were evaluated by RT-PCR. Phospo-NF-κB was evaluated in total homogenates and in the nucleus fraction by Western blot; TLR4, COX-2, RAGE and Iba1 protein expression were also determined in total homogenates. LPS increases only the immunocontent of the pro-inflammatory IL-1β in the intracellular slices (**A**) and augments its secretion to the extracellular medium (**B**). Only gene expression of ILR1 was reduced by LPS incubation (**C**), and there were no changes in the expressions of genes encoding IL-1β, and TNF-α and its receptor TNFR1. Acute inflammation promotes elevation of RAGE and COX2 protein expression (**D**) and translocation to the nucleus of phospho-NF-κB (**E**). Values are expressed as means ± standard error. Data were analyzed by Student's unpaired t test, assuming *P* < 0.05. * means significant increase, when compared to sham group, # means significant decrease, when compared to sham group

In order to assess the early effects of neuroinflammation on the glycolytic pathway, a further set of ex vivo experiments was carried out. One hour of acute LPS exposure in hippocampal slices was sufficient to increase the tissue IL-1β immunocontent (Fig. 5A, *P* = 0.0175) and IL-1β secretion (Fig. 5B, *P* = 0.0191), without changing cellular integrity (Additional file 4: Figure S4), followed by a decrease in ILR1 mRNA levels (Fig. 5C, *P* = 0.03432). LPS did not affect the intra and extracellular immunocontents of TNF-α (Fig. 5A, B, *P* = 0.944 and *P* = 0.8681), nor the gene expressions of IL1b, TNF-α and TNFR1 (Fig. 5C). The elevation of IL-1β cytokine levels was associated with increased cytosol inflammasome assembly and we detected an increase in NLRP3 gene expression (Fig. 5D, *P* = 0.0270). Furthermore, we confirmed that this acute model activated other mediators of neuroinflammation signaling in the hippocampus, as LPS increased both the mRNA (Fig. 5D, *P* = 0.0050) and protein (Fig. 5E, *P* = 0.0290) levels of the RAGE receptor. This was followed by an augmentation of the inducible enzyme, cyclooxygenase-2 (COX2) (Fig. 5E, *P* = 0.0255), and nuclear translocation of the NF-κB transcription factor (Fig. 5F, *P* = 0.0476).

**Fig. 6:**
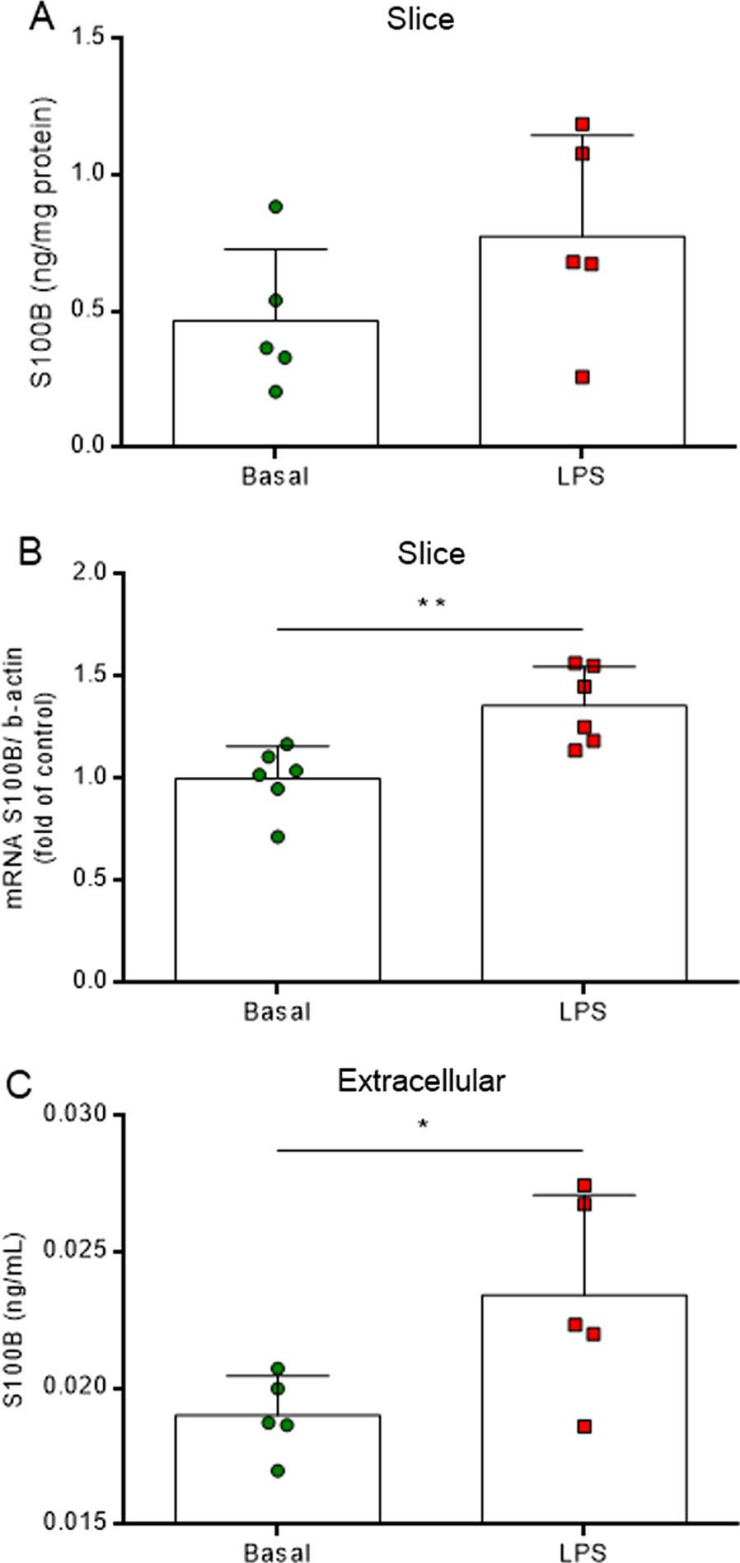
LPS induces astrocyte reactivity in acute hippocampal slices. S100B was measured by ELISA and S100B gene expression was evaluated by RT-PCR. LPS did not change the intracellular immunocontent of S100B protein (**A**); however, it did upregulate S100B gene expression (**B**) and elevate S100B secretion (**C**). Values are expressed as means ± standard error. Data were analyzed by ANOVA, followed by the Tukey test, assuming *P* < 0.05. * means significant increase, when compared to sham group (* *P* < 0.05, ** *P* < 0.01)

Acute neuroinflammation also affected astrocytes and glutamate flow in the ex vivo hippocampal slices model. We observed a decrease in S100B gene expression (Fig. 6B, *P* = 0.0053), without changes in protein levels (Fig. 6A, *P* = 0.1647), and an elevation of S100B secretion (Fig. 6C, *P* = 0.0366). LPS also promoted impairment of the glutamate system by reducing glutamate uptake. This imbalance occurred independently of changes in the expressions of the glutamate transporter proteins, GLAST and GLT1. As a consequence of extracellular glutamate elevation, we observed an excitotoxicity glutamatergic event, which resulted in high protein levels of NMDAR1 and oxidative stress, through a reduction in GSH content and the generation of total reactive oxygen species (DCF) (Additional file 5: Figure S5).

### LPS induces changes in the glycolysis pathway

**Fig. 7:**
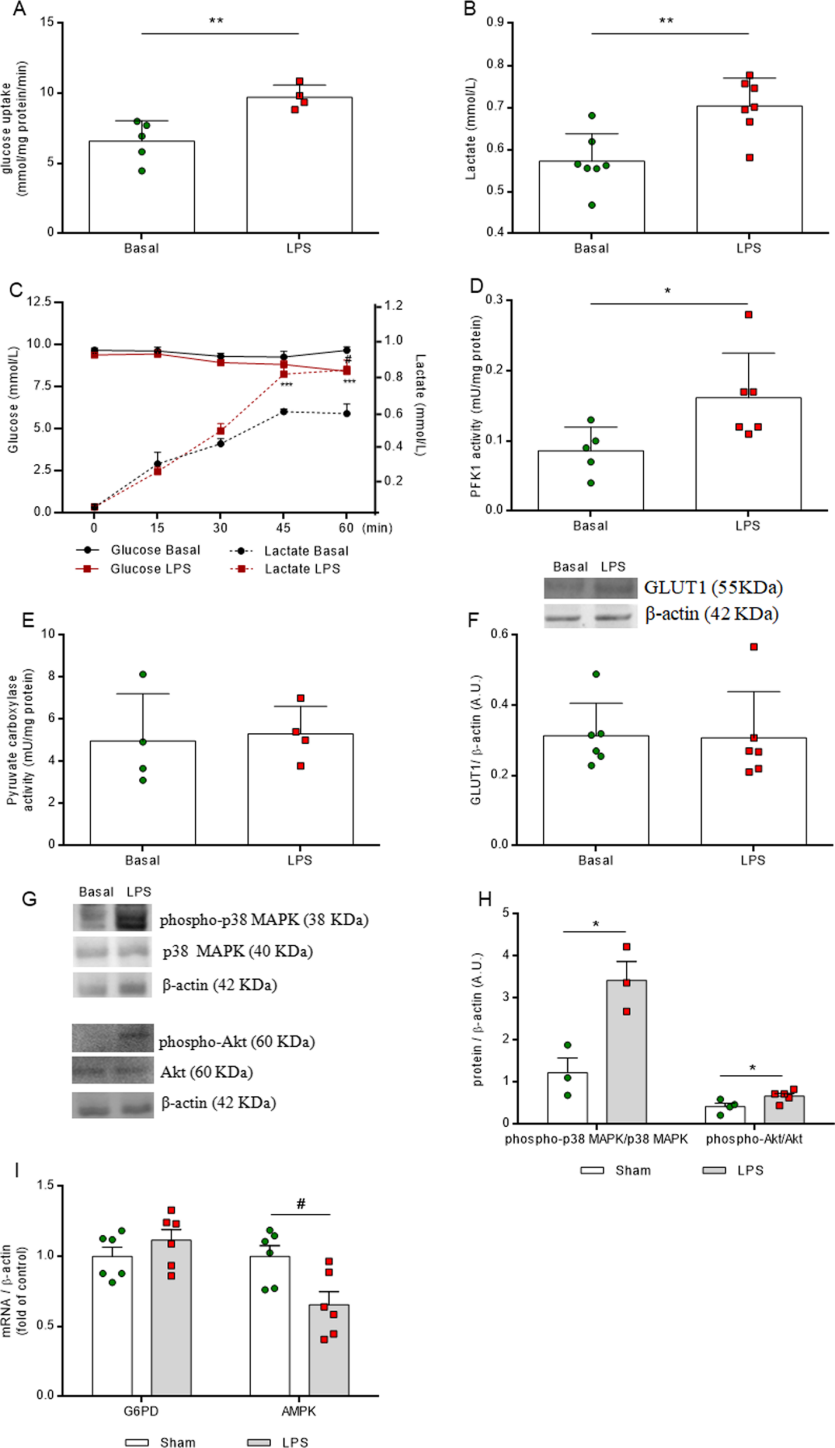
LPS induces glycolytic pathway activity in acute hippocampal slices. Glucose uptake was measured by a radioactivity assay. Glucose and lactate medium levels were evaluated by a spectrophotometric method. Pyruvate carboxylase and PFK1 activities were analyzed by kinetic assay. Protein expressions of the glucose transporter (GLUT1) and phosphorylation of p38 MAPK and Akt were analyzed by Western blot. Gene expressions of the enzymes G6PD and AMPK were evaluated by RT-PCR. Inflammatory induction increased glucose uptake in hippocampal slices (**A**) and lactate extracellular levels (**B**). There was also a time-dependent release of lactate to the extracellular medium and a decrease of glucose content in the medium (C). LPS increases PFK1 activity (D), without changing pyruvate carboxylase activity (**E**) or changing GLUT1 transporter protein expression (**F**). Representative image of Western blot (**G**). Early inflammation induces phosphorylation of key enzymes related to energy metabolism, p38MAPK and Akt (H). The gene expression of G6PD was not affected (**I**), rather AMPK gene expression was downregulated (I). Data were analyzed by Student's unpaired t test, assuming *P* < 0.05. * means significant increase, when compared to sham group (* *P* < 0.05, ** *P* < 0.01), # means significant decrease

In further experiments, the astrocytic response to the inflammatory stimulus was found to generate an increase in glucose uptake in hippocampal slices (Fig. 7A, *P* = 0.0071). This augmentation in glucose uptake occurred via an elevation in glucose consumption, leading to lower extracellular glucose levels within 60 min (Fig. 7C, *P* = 0.0141), followed by a time-dependent production and release of lactate to the extracellular medium after 45 min of incubation (Fig. 7C, *P* < 0.0001). Glycolysis pathway acceleration was observed at 60 min of inflammation induction, in association with high levels of lactate in the extracellular medium (Fig. 7B, *P* = 0.0029), which correlated with an increase in PFK1 activity (Fig. 7D, *P* = 0.0412), without changes in pyruvate carboxylase activity (Fig. 7E, *P* = 0.8037). These metabolic changes occurred independently of GLUT1 transporter protein expression (Fig. 7F, *P* = 0.9301); however, they were able to induce phosphorylation of key metabolic enzymes, such as p38MAPK (Fig. 7G, H, *P* = 0.0179) and Akt (Fig. 7G, H, *P* = 0.0449). The gene expression of G6PD was not affected (Fig. 7I, *P* = 0.2792), but AMPK gene expression was downregulated (Fig. 7I , *P* = 0.0169).

Following observations that our acute hippocampal slice model generated neuroinflammation and changes in astrocyte energy metabolism, we investigated the effects of the inhibition of the glycolytic enzymes (hexokinase, PFKFB3 and LDH), as well as of the Krebs cycle, enzyme aconitase, on changes in glucose uptake and PFK1 activity, and in the content and secretion of cytokine IL-1β and S100B. Subsequently, we downregulated inflammatory signaling and evaluated effects on energy metabolism, cytokine IL-1β and S100B protein. For this purpose, we inhibited the activation of microglia and astrocyte cells, as well as the assembly of the NLRP3 inflammasome. None of the inhibitors (metformin, 3PO, oxamic acid, fluorocitrate, arundic acid, minocycline or MCC950) changed the cellular integrity of the hippocampal slices (Additional file 4: Figure S4).

**Fig. 8:**
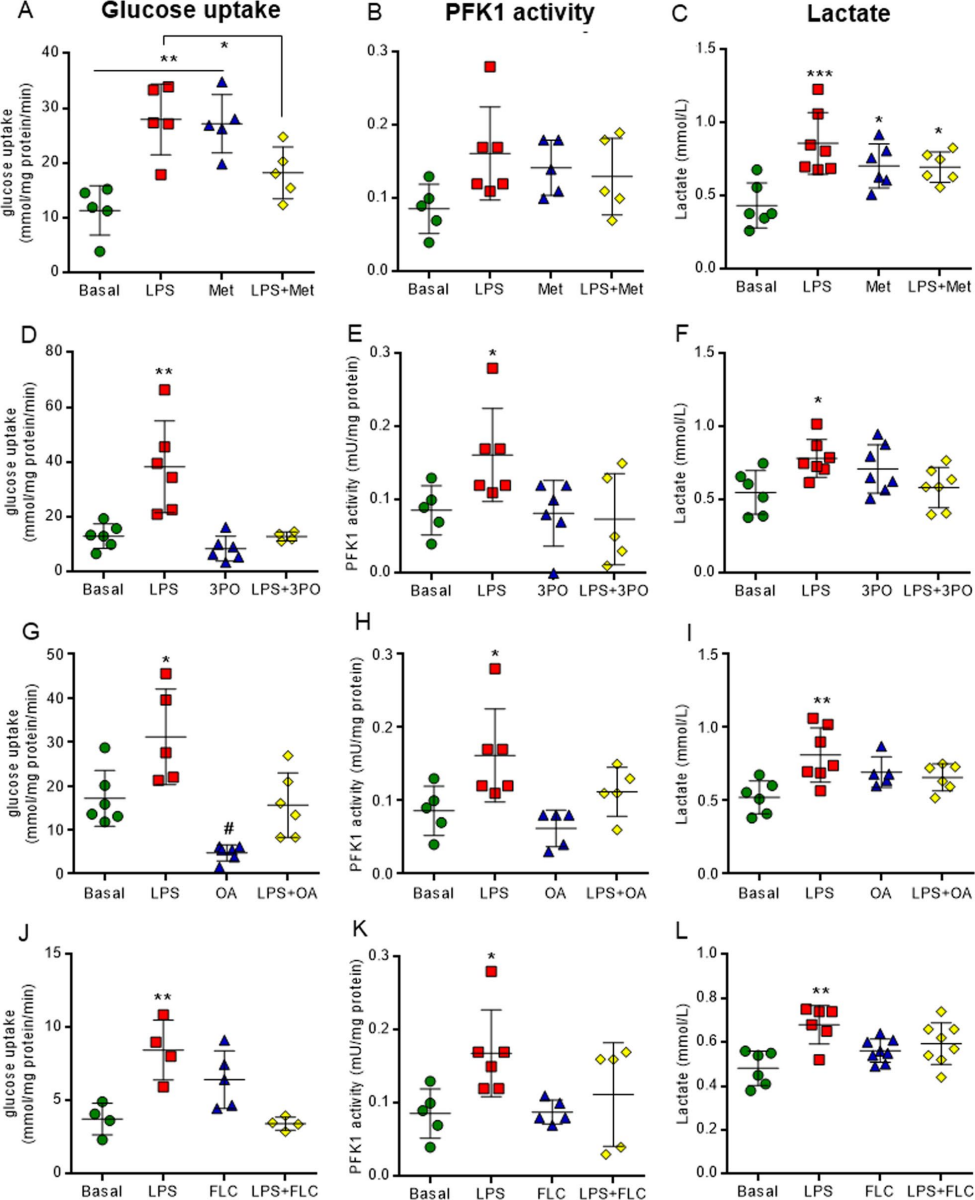
Inhibitors of energetic metabolism reverse the effects of LPS on the glycolytic pathway in acute hippocampal slices. Glucose uptake was measured by a radioactivity assay. PFK1 activity was analyzed by kinetic assay. Lactate medium levels were evaluated by a spectrophotometric method. Metformin, a non-specific hexokinase inhibitor, increased glucose uptake per se, however it reversed the effects of LPS during co-incubation (**A**). Metformin per se increased extracellular lactate levels (**C**), without changing PFK1 activity (**B**) and did not affect such changes induced by LPS. The inhibitor of PFK1 (3PO), LDH (oxamic acid, OA), and aconitase (fluorocitrate, FLC) reversed the neurometabolic changes induced by LPS (**D**-**L**). Values are expressed as means ± standard error. Data were analyzed by ANOVA, followed by the Tukey test, assuming *P* < 0.05. *means significant increase when compared to sham group

**Fig. 9:**
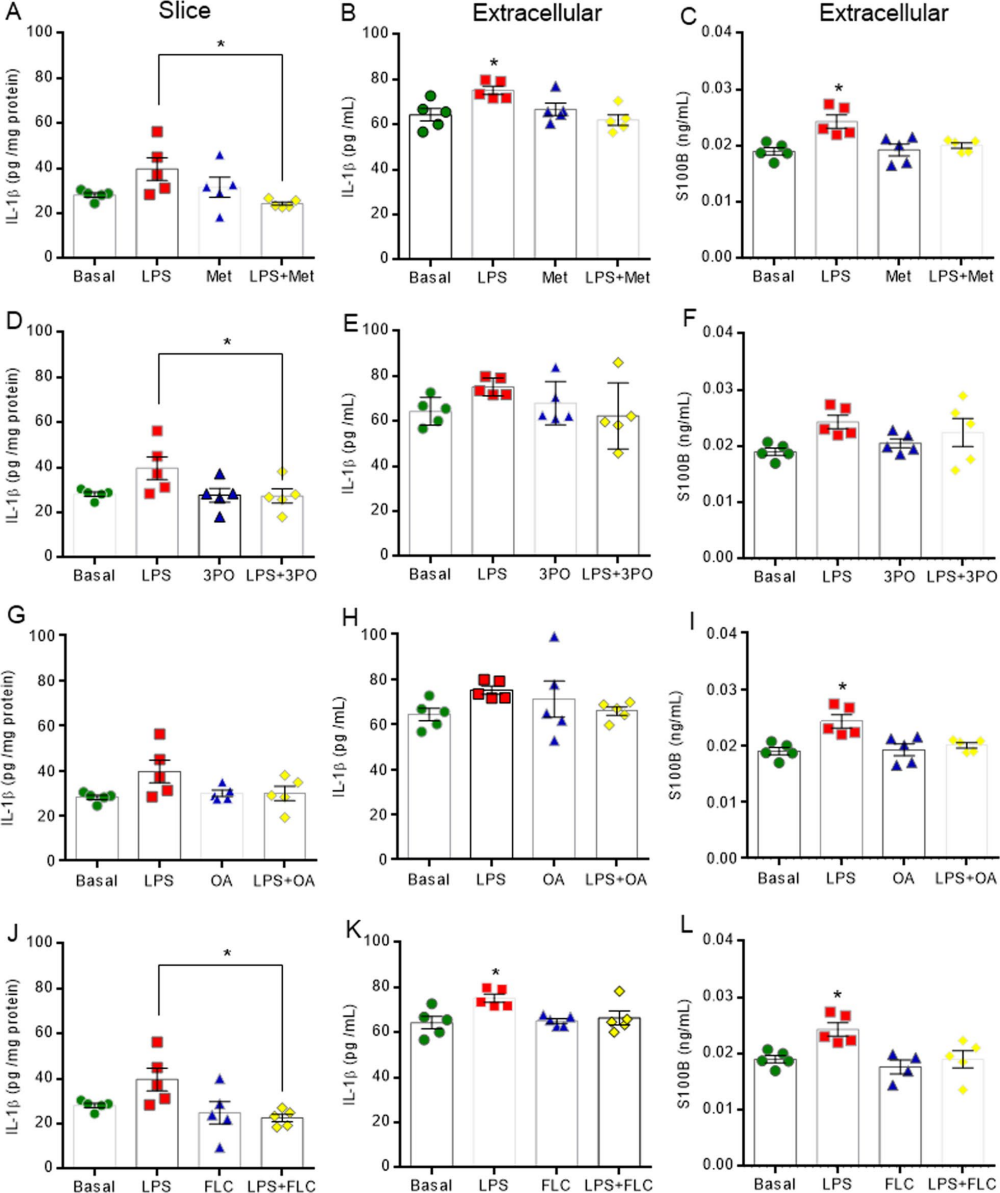
Inhibitors of energy metabolism reverse the neuroinflammation induced by LPS in acute hippocampal slices. Pro-inflammatory immunocontents of IL-1β and the S100B protein were measured by ELISA. Metformin, a non-specific hexokinase inhibitor, decreases intra (**A**) and extracellular (**B**) IL-1β levels, as well as S100B secretion (**C**). The inhibitor of PFK1 (3PO) reverses effect induced by LPS on the intracellular cytokine (**D**), without changes in the secretion of IL-1β (**E**) or S100B (**F**). The inhibitor of LDH, oxamic acid (OA) only reverses LPS-induced S100B secretion (**I**). Conversely, the inhibitor of aconitase (fluorocitrate, FLC) reverses the LPS-induced inflammatory effect on the secretion of IL-1β (**K**) and of S100B (**L**), and decreases intracellular IL-1β (**J**). Values are expressed as means ± standard error. Data were analyzed by ANOVA, followed by the Tukey test, assuming *P* < 0.05. *means significant increase, when compared to sham group

The inhibitors of the glycolytic pathway and of aconitase (Krebs cycle enzyme) reversed the changes in neurometabolism induced by LPS (Fig. 8). The inhibitors of PFKFB3 (3PO) (Fig. 8D, E, *F*_(3,18)_ = 12.130, *P* = 0.0001 and F_(3,18)_ = 3.455, *P* = 0.0384), LDH (oxamic acid) (Fig. 8G, H, *F*_(3,19)_ = 12.610, *P* = 0.0001 and F_(3,17)_ = 5.416, *P* = 0.0084) and aconitase (fluorocitrate) (Fig. 8J, K, *F*_(3,13)_ = 9.290, *P* = 0.0015 and F_(3,17)_ = 3.288, *P* = 0.0461) also reversed the high glucose uptake and PFK1 activity, induced by neuroinflammation. Moreover, they returned extracellular lactate content to basal levels (Fig. 8D, I and L, *F*_(3,23)_ = 3.707, *P* = 0.0260, F_(3,20)_ = 5.092, *P* = 0.0088 and F_(3,24)_ = 6.431, *P* = 0.0024). The non-specific regulator of hexokinase (metformin) per se increased glucose uptake and lactate levels without changes when co-incubated with LPS (Fig. 8A, C, *F*_(3,16)_ = 11.060, *P* = 0.0004 and *F*_(3,21)_ = 7.493, *P* = 0.0014). The modulation of key enzymes of energetic metabolism also promoted changes in neuroinflammation signaling (Fig. 9) and oxidative stress induction (Additional file 6: Figure S6). Metformin decreased intra and extracellular IL-1β levels, as well as S100B secretion (Fig. 9A–C, *F*_(3,16)_ = 3.673, *P* = 0.0347, *F*_(3,16)_ 5.468, *P* = 0.0088 and *F*_(3,16)_ = 7.884, *P* = 0.0019, respectively). The down-regulation of PFK1, achieved by inhibition of PFKFB3, reversed the effects of LPS on intracellular cytokine protein (Fig. 9D, *F*_(3,16)_ = 3.115, *P* = 0.0500), in contrast to the inhibitor of LDH which reversed only the induction of S100B secretion (Fig. 9I_(3,16)_ = 7.884, *P* = 0.0019). The inhibition of aconitase (Krebs cycle enzyme) reversed the LPS-induced elevations in IL-1β and S100B secretion and the increase in the intracellular immunocontent of IL-1β (Fig. 9J-L, *F*_(3,16)_ = 4.285, *P* = 0.0212, F_(3,16)_ = 4.626, *P* = 0.0163 and F_(3,16)_ = 6.288, *P* = 0.0056, respectively).

**Fig. 10:**
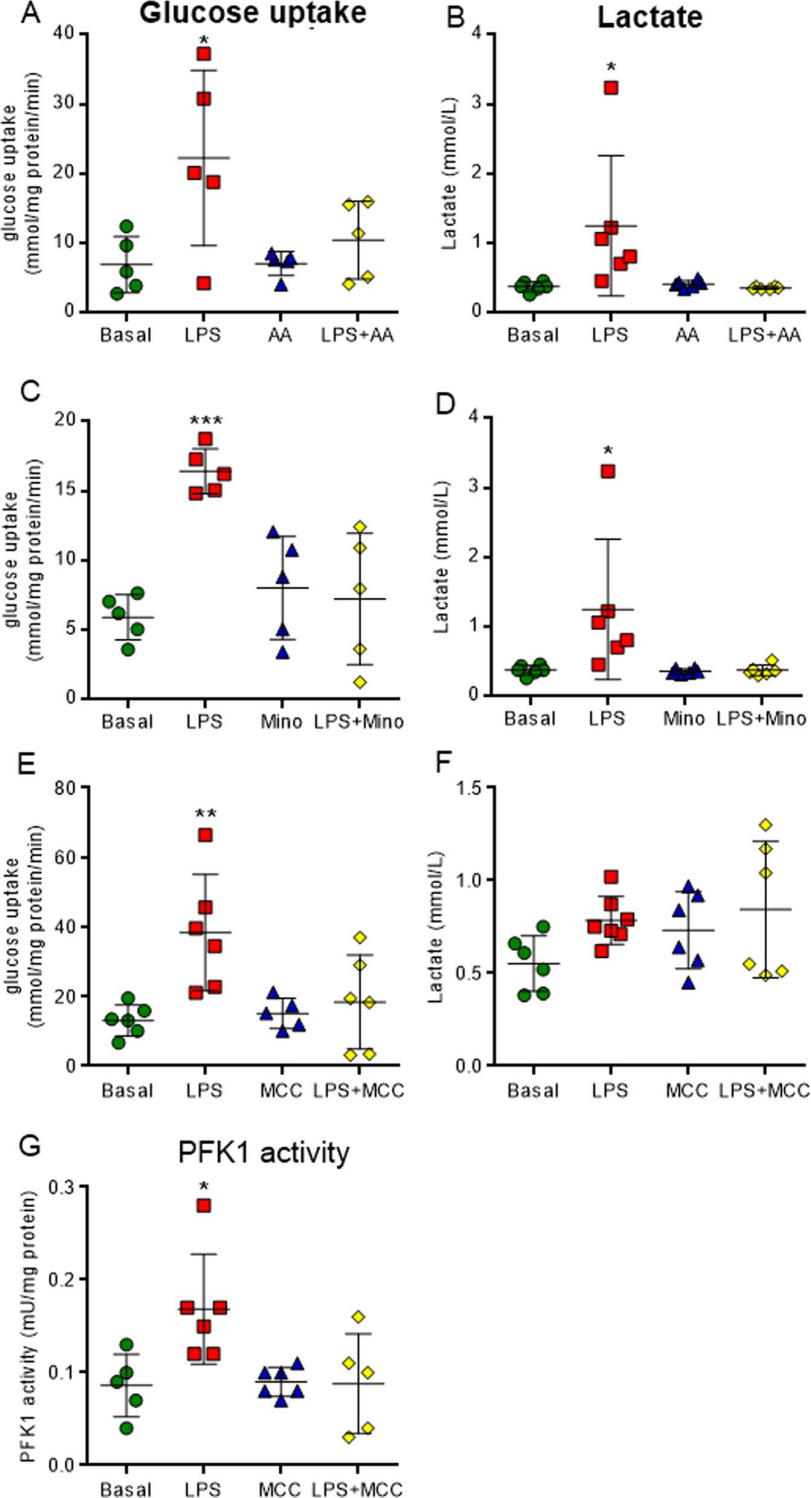
Inhibitors of neuroinflammation signaling reverse the changes in the glycolytic pathway induced by LPS in acute hippocampal slices. Glucose uptake was measured by a radioactivity assay. Lactate medium levels were evaluated by a spectrophotometric method. PFK1 activity was analyzed by kinetic assay. Downregulation of neuroinflammation reverses the glucose uptake effects. The effects of arundic acid (AA), an inhibitor of reactive astrocytes (**A**), minocycline (mino), an inhibitor of microglia cells (**C**), and MCC950 (MCC), an inhibitor of NLRP3 assembly (**E**), on glucose uptake were evaluated. The high lactate extracellular levels were reversed by AA (**B**) and mino (**D**). The specific inhibition of NLRP3 reverses the activity of PFK1 (**G**). Values are expressed as means ± standard error. Data were analyzed by ANOVA, followed by the Tukey test, assuming *P* < 0.05. * means significant increase when compared to sham group

**Fig. 11:**
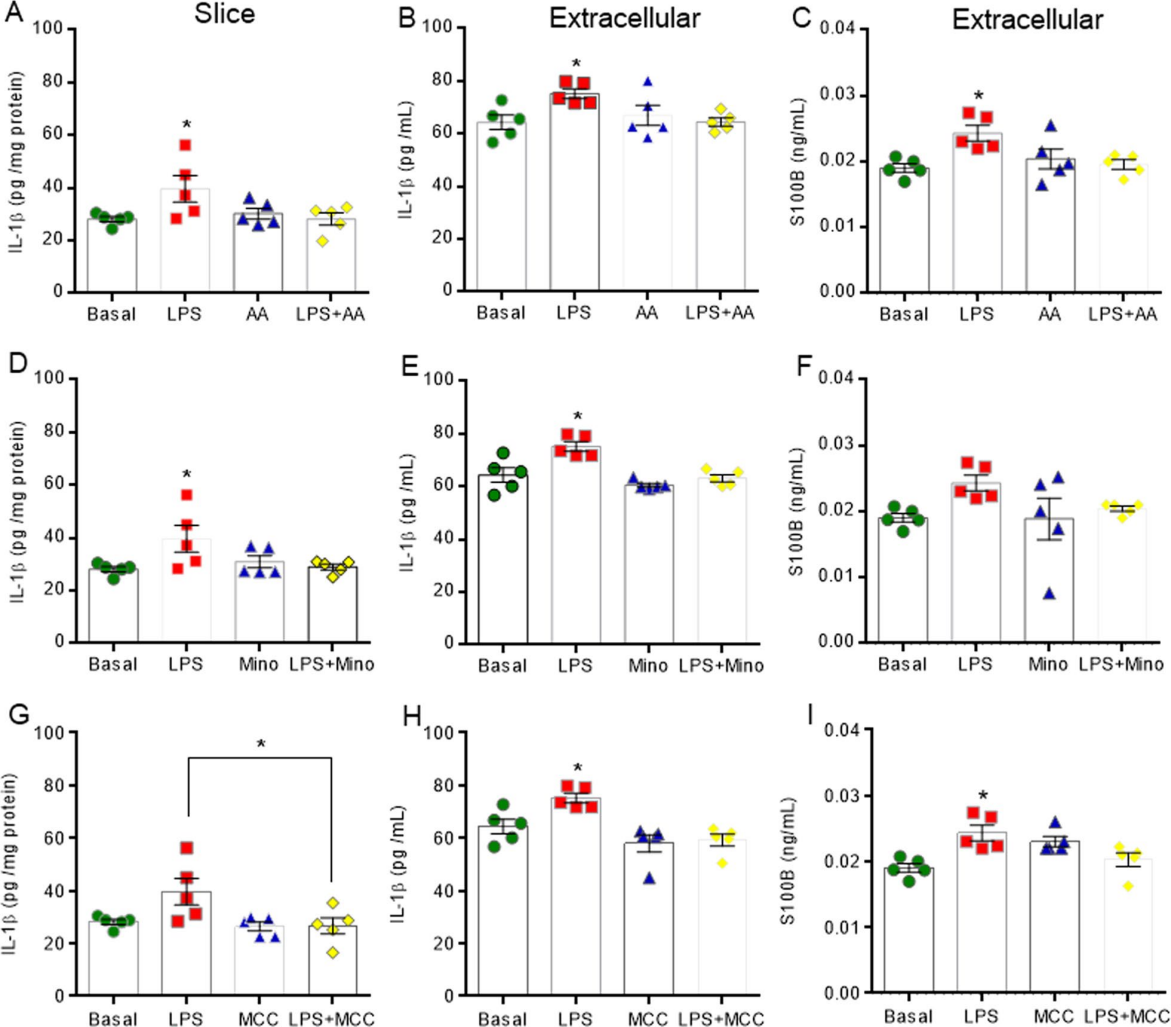
Inhibitors of neuroinflammation signaling reverse the effects of LPS on IL-1β and S100B in acute hippocampal slices. Pro-inflammatory immunocontents of IL-1β and the S100B protein were measured by ELISA. Astrocyte inhibition by arundic acid (AA) reversed the elevations in IL-1β and S100B (**A**-**C**). Microglia inhibition by minocycline (mino) only reversed the increase in IL-1β (**D**-**F**). NLRP3 inhibition by MCC950 (MCC) reversed the IL-1β and S100B secretion that was induced by LPS (**H**, **I**) and, when compared to LPS group, the intracellular immunocontent of IL-1β (**G**). Values are expressed as means ± standard error. Data were analyzed by ANOVA, followed by the Tukey test, assuming *P* < 0.05. * means significant increase, when compared to sham group

Interestingly, the inhibition of neuroinflammation signaling reversed the LPS-induced alterations in glycolytic parameters (Fig. 10). The inhibitors of astrocytes (arundic acid), microglia (minocycline) and NLRP3 (MCC950) also reversed the effect of inflammation on glucose uptake (Fig. 10A, C and E, F_(3,16)_ = 4.981, *P* = 0.0125, F_(3,16)_ = 11.000, *P* = 0.0004 and F_(3,16)_ = 6.065, *P* = 0.0045, respectively). The high extracellular lactate levels that were induced by LPS were reversed by the inhibition of microglia and astrocytes (Fig. 10B and D, F_(3,16)_ = 4.403, *P* = 0.0156 and F_(3,16)_ = 4.49, *P* = 0.0144, respectively). In particular, the inhibition of NLRP3 returned PFK1 activity to its basal level (Fig. 10G, F_(3,16)_ = 4.864, *P* = 0.0019). These molecules generally reverse the effects of LPS on pro-inflammatory cytokine IL-1β and S100B secretion (Fig. 11), and on oxidative stress induction (Additional file 6: Figure S6). Astrocyte inhibition reversed the effects induced by inflammation on IL-1β and S100B (Fig. 11A–C, F_(3,16)_ = 3.371, *P* = 0.00447, F_(3,16)_ = 3.716, *P* = 0.0335 and F_(3,16)_ = 5.407, *P* = 0.0119, respectively), whereas down modulation of microglia only affected cytokine IL-1β (Fig. 11D-E, F_(3,16)_ = 3.383, *P* = 0.0442 and F_(3,16)_ = 12.760, *P* = 0.0002, respectively). The inhibition of NLRP3 reversed the LPS-induced IL-1β and S100B secretion and elevation in intracellular immunocontent of IL-1β that was observed in hippocampal slices (Fig. 11G-I, F_(3,16)_ = 4.209, *P* = 0.0225, F_(3,16)_ = 9.192, *P* = 0.0090 and F_(3,16)_ = 6.848, *P* = 0.0035, respectively).

## Discussion

Neuroinflammation is a pathophysiological event that is common to many neurological disorders, such as traumatic brain injuries and neurodegenerative diseases, which are characterized by extensive functional changes in brain cells, including glial cells [35]. Neurodegeneration is known to be related to events of neuroinflammation, astrocytic reactivity and dysfunction [4, 36]. In this study, we used two models of neuroinflammation in order to access the effects of neuroinflammation on hippocampal glycolytic metabolism. In the first in vivo model, neuroinflammation was evaluated at 6 and 24 h after the *i.p.* injection of LPS, and the time-dependent neurometabolism changes were monitored. In a second model, an ex vivo acute neuroinflammation model was induced in hippocampal slices by incubating slices with LPS for 1 h; and the modulation of the glycolysis pathway was then determined. It is well known that the Gram-negative bacteria endotoxin, LPS, employed in our study, stimulates glial cells and induces neuroinflammation in rodents (when administrated intraperitoneally) [18] and in acute hippocampal slices [21]. Our data also confirm that LPS is able to induce neuroinflammation activation in both of these models. It is worth mentioning that, under our conditions, the alterations in IL-1β in response to LPS exposure were more reliable than those of TNF-α, both in vivo and in vitro.

We observed, in vivo, an increase in the hippocampal content of IL-1β and nuclear translocation of the NF-κB transcription factor at both 6 and 24 h after LPS injection, with elevated inflammasome NLRP3 gene expression only at 6 h. The NLRP3 inflammasome cleaves the IL-1β precursor to its mature pro-inflammatory cytokine form for subsequent secretion [37], possibly contributing to the maintenance of high levels of the IL-1β cytokine during the early neuroinflammatory response, and promoting neuronal dysfunction and/or death due to glutamatergic excitotoxicity [9]. In fact, our results suggest that glutamatergic excitotoxicity is associated with the early neuroinflammatory response, where glutamate uptake is reduced, leading to oxidative stress (due to low levels of GSH, non-enzymatic antioxidant defense). LPS-induced neuroinflammation also alters astrocyte reactivity in vivo. At 6 h, the detection of high levels of the cytoskeleton protein GFAP is indicative of astrogliosis [38]. Moreover, at both times evaluated, we observed an increase in the levels of S100B in the CSF, which is secreted predominantly by astrocytes. The extracellular S100B protein exerts a paracrine action on neurons and microglia, and an autocrine action on astrocytes [39]. In the extracellular milieu, S100B signals occur in a dual manner, depending on its concentration. At nanomolar concentrations, it acts as a neurotrophic factor, promoting neurite outgrowth, synaptic modulation and neuronal survival [40]. However, at high concentrations, S100B acts as a damage-associated molecular pattern (DAMP) [41] and promotes neurotoxic effects, triggering activation of tissue injury signaling, inflammatory responses and apoptosis [40]. Herein, we consider the increase in S100B levels in the CSF to be a biomarker of the tissue damage promoted by LPS-induced neuroinflammation, as also observed in previous studies [21, 42].

We observed that early neuroinflammation signaling promotes metabolic reprogramming in the hippocampus. At 6 h after *i.p.* LPS administration, an increase in hippocampal glucose uptake was observed, accompanied by a high lactate content and low levels of glucose in the CSF. At this same time point, we did not observe any changes in glutamate uptake or mitochondria activity, which suggests greater modulation of the glycolysis pathway in order to promote and maintain the synthesis and secretion of mediators related to early neuroinflammation signaling. It is known that glutamate uptake by astrocytes is mediated by a Na^+^ dependent gradient [43] and associated with higher glucose consumption [31, 44]. Our data do not suggest this correlation between glucose and glutamate uptake, and indicate that the glycolytic pathway is used to provide energy support to perform the inflammatory response. Furthermore, previous studies on pro-inflammatory/oxidative stress induction have demonstrated a reduction in respiratory capacity and a shift in metabolism to the glycolytic pathway in peripheral innate cells, such as macrophages [17, 45], and in innate nervous cells, such as microglia [46–48] and astrocytes [14, 49, 50]. It is worth mentioning that this in vivo neurometabolic reprogramming should be further studied and that other glycolytic parameters should be included in future studies. Furthermore, it should be taken into account that the mitochondrial changes that occurred in the 24-h period evaluated may represent either a compensation or a dysfunction effect, or that they occur due to problems in energy supply or a reduction in glycolysis per se. As such, we went on to further investigate the early effects of neuroinflammation on glycolysis pathway using an ex vivo hippocampal slice model.

Our results provide further evidence of the occurrence of neuroinflammation and the induction of astrocyte reactivity in acute hippocampal slices. The incubation of these slices with LPS promoted the upregulation of neuroinflammatory signaling, accompanied by changes in intra and extracellular pro-inflammatory IL-1β immunocontent, RAGE and NLRP3 gene expression, RAGE and COX2 protein expression and NF-κB translocation to the nucleus, without changes in the immunocontent of Iba1, the microglia biomarker. Interestingly, in this acute LPS-induced model, astrocyte reactivity predominated rather than microglial activity, as indicated by increases in the gene expression and secretion of S100B. Our results in acute hippocampal slices corroborate reports of a neurotoxic effect of this protein in the extracellular milieu [24, 42], since the induction of acute neuroinflammation promoted an increase in S100B secretion, leading to glutamatergic excitotoxicity via a reduction in glutamate uptake and due to oxidative stress.

A metabolic shift to glycolysis was also observed in this acute neuroinflammation model, associated with a reduction in glutamate uptake, suggesting again that the increase in glucose consumption is not directed to glutamate homeostasis, but provides energy support for the maintenance of the inflammatory response. We, herein, considered glucose uptake, PFK1 activity and extracellular lactate levels as glycolytic parameters; when we modulated some key enzymes of glycolysis and the Krebs cycle, the effects of neuroinflammation on these parameters were reversed. Metformin, 3PO, and oxamic acid are inhibitors of hexokinase (non-specific) [51, 52], PFKFB3 (which decreases the positive allosteric modulator of PFK1 enzyme, fructose-2,6-bisP) [53–55] and LDH [56–58], respectively. Fluorocitrate is an inhibitor of aconitase, which is an enzyme of the Krebs cycle [24, 59]. Interestingly, only metformin increased glucose uptake per se; metformin is an anti-glycemic agent and has been used for the treatment of type II diabetes. Evidence in peripheral tissue suggests that metformin alters the intracellular redox state through the inhibition of mitochondrial glycerol-3-phosphate dehydrogenase (and thereby indirectly affects LDH activity) [60]. This energy imbalance may also occur in nervous tissue and affect glucose consumption, resulting in an elevation in glucose uptake. Accordingly, previous studies have suggested that metformin increases the glycolytic flux in astrocyte cultures by enhancing glucose consumption and lactate release, thereby reducing Krebs cycle activity and mitochondrial complex I activity [61–63]. The down-regulation of the glycolytic pathway also affects neuroinflammatory signaling, reinforcing the role of this metabolic pathway in the inflammatory response. The main metabolic pathway enzyme inhibitors used, with the exception of oxamic acid, clearly reversed the activation of the inflammatory pathway, reducing IL-1β synthesis and secretion, and decreasing S100B protein secretion.

In order to reinforce the effect of neuroinflammation in glycolysis pathway activation, we employed inhibitors of inflammation or glial cells reactivity. Arundic acid is considered an inhibitor of S100B synthesis and secretion [25, 64] which leads a down-regulation of astrocyte reactivity. Minocycline affects microglia cells [65, 66], thereby inhibiting their cross-talk with astrocytes during neuroinflammation as a whole. Additionally, MCC950 inhibits NLRP3 inflammasome assembly and affects the maturation and secretion of the pro-inflammatory cytokine, IL-1β [67, 68]. Our results demonstrated that these inhibitors reversed the augmentations in glucose uptake and lactate levels that were induced by LPS. In particular, the inhibition of NLRP3 assembly reversed PFK1 activity. In addition, as a control, we also observed the actions of these inhibitor molecules on neuroinflammatory signaling, observing the reversal of the LPS-induced effects on IL-1β and S100B.

## Conclusion

Our data demonstrate temporal changes in neurometabolism following the induction of neuroinflammation by LPS, in vivo. Early neuroinflammation (6 h after LPS) promoted a shift in energy metabolism, to increase glycolysis. Consistent with this finding, acute inflammation also promoted metabolic reprogramming in hippocampal slices, enhancing glucose uptake, PFK1 activity and extracellular lactate levels; these changes were considered to be glycolytic parameters, and the neurometabolism shift event observed was similar to that of the Warburg effect. Accordingly, the modulation of key enzymes of glycolytic metabolism and neuroinflammation reduced IL-1β and S100B secretion. In addition, the inhibition of S100B (a protein predominantly synthesized and secreted by astrocytes), microglia activation inhibition and abrogation of the assembly of NLRP3 inflammasome confirmed the roles of neuroinflammation in the upregulation of glycolysis in the hippocampus. Finally, our data suggest that key steps in glycolysis may represent promising pharmacological targets for downregulating the effects of neuroinflammation, a common feature of neurodegenerative diseases and neurological disorders.

## Supplementary Information

**Additional file 1: Figure S1.** Dose curve of the effects of glycolytic pathway inhibitors on glucose uptake. Glucose uptake was measured by a radioactivity assay. Lactate medium levels were evaluated by a spectrophotometric method. Dose of 20 μM of 3PO reduces glucose uptake (A), without changing extracellular lactate levels (B). Doses of 10 and 50 μM of oxamic acid (OA) decrease glucose uptake (C), without changing extracellular lactate levels (D). Values are expressed as means ± standard error. Data were analyzed by ANOVA, followed by the Tukey test, assuming *P* < 0.05. # means significant decrease, when compared to sham group.

**Additional file 2: Figure S2.** LPS activates peripheral inflammation. Serum immunocontents of TNF-α and S100B were measured by ELISA. Glucose and lactate serum levels were evaluated by spectrophotometric method. TNF-α (A) and S100B (B) immunocontent serum levels are increased only at 6 h after LPS. Serum glucose levels decreased at both time points (C). Serum lactate levels increased only at 6 h Values are expressed as means ± standard error. Data were analyzed by ANOVA, followed by the Tukey test, assuming *P* < 0.05. * means significant increase, when compared to sham group (* *P* < 0.05, ** *P* < 0.01), # means significant decrease, when compared to sham group.

**Additional file 3: Figure S3.** LPS induces glutamatergic neurotoxicity in the hippocampus. Glutamate uptake was measured by a radioactivity assay. GSH content and total reactive oxygen species (DCF) were measured by a fluorescent method. Administration of LPS decreases glutamate uptake only at 6 h (A). Both time points of neuroinflammation induction reduce non-enzymatic antioxidant defense, GSH content (B), without changes in DCF production (C). Values are expressed as means ± standard error. Data were analyzed by ANOVA, followed by the Tukey test, assuming *P* < 0.05. # means significant decrease, when compared to sham group.

**Additional file 4: Figure S4.** LPS promotes glutamatergic neurotoxicity in acute hippocampal slices. Glutamate uptake was measured by a radioactivity assay. Protein expressions of glutamate receptor and glutamate transporters (GLAST, GLT1) were analyzed by Western blot. GSH content and total reactive oxygen species (DCF) were measured by a fluorescent method. LPS decreases glutamate uptake (A), regardless of the expressions of the GLAST and GLT1 transporters (B). Neuroinflammation increases NMDAR1 expression (B) and oxidative stress by reducing the non-enzymatic antioxidant defense, GSH content (C), and increases DCF production (D). Values are expressed as means ± standard error. Data were analyzed by Student's unpaired t test, assuming *P* < 0.05. * means significant increase, when compared to sham group, # means significant decrease, when compared to sham group (# *P* < 0.05, ## *P* < 0.01).

**Additional file 5: Figure S5.** LPS and inhibitors of energetic metabolism and neuroinflammation do not change cellular integrity in hippocampal slices. Extracellular LDH activity was evaluated by a spectrophotometric method. Incubation of hippocampal slices with the following for one hour in the presence of LPS (10 μg/mL); (A), Metformin (Met, 500 μM) (B), 3PO (20 μM) (C), oxamic acid (OA, 10 μM) (D), fluorocitrate (FLC, 10 μM) (E), arundic acid (AA, 100 μM) (F), minocycline (Mino,10 μM) (G), MCC950 (MCC, 10 μM) (H), or co-incubation, did not alter cellular integrity. Values are expressed as means ± standard error. Data were analyzed by ANOVA, followed by the Tukey test, assuming *P* < 0.05.

**Additional file 6: Figure S6.** Effects of metabolic and neuroinflammation inhibitors on LPS-induced oxidative stress in acute hippocampal slices. GSH content and total reactive oxygen species (DCF) were measured by a fluorescent method. The inhibitors 3PO (20 μM) (A-B), fluorocitrate (FLC, 10 μM) (C-D), arundic acid (AA, 100 μM) (E–F), minocycline (Mino,10 μM) (G-H), and MCC950 (MCC, 10 μM)(I-J) reversed the reduction in GSH content and elevation in total reactive oxygen species (DCF) promoted by LPS. Values are expressed as means ± standard error. Data were analyzed by Student's unpaired t test, assuming *P* < 0.05. * means significant increase, when compared to sham group, # means significant decrease, when compared to sham group (# *P* < 0.05, ## *P* < 0.01).

**Additional file 7: Table S1.** List of antibodies used in this study.

## Data Availability

The data sets used and/or analyzed during the current study are available from the corresponding author on reasonable request.

## Abbreviations

DCF: 2′7’-Dichlorofluorescein
3PO (): 3-(3-Pyridinyl)-1-(4-Pyridinyl)-2-Propen-1-One
AA: Arundic acid
COX2: Cyclooxygenase2
FLC: Fluorocitrate
GFAP: Glial fibrillary acidic protein
GLUT1: Glucose transporter 1
EAAT2 and EAAT1: Glutamate transporters
IL-1β: Interleukin 1 beta
i.p.: Intraperitoneal
Iba1: Ionized calcium-binding adapter molecule 1
LDH: Lactate dehydrogenase
LPS: Lipopolysaccharide
MCC: MCC950
Met: Metformin
Mino: Minocycline
NLRP3: NLR family pyrin domain containing 3
OA: Oxamic acid
PFK1: Phosphofructokinase-1
RAGE: Receptor of advanced glycation end-products
GSH: Reduced glutathione
S100B: S100 calcium-binding protein B
TLR4: Toll-like receptor 4
TNF-α: Tumor necrosis factor
