# Supplementary figures and images for: Early effects of LPS-induced neuroinflammation on the rat hippocampal glycolytic pathway
Source: J Neuroinflammation. 2022 Oct 11;19:255. doi: 10.1186/s12974-022-02612-w (PMC9552490; doi:10.1186/s12974-022-02612-w)

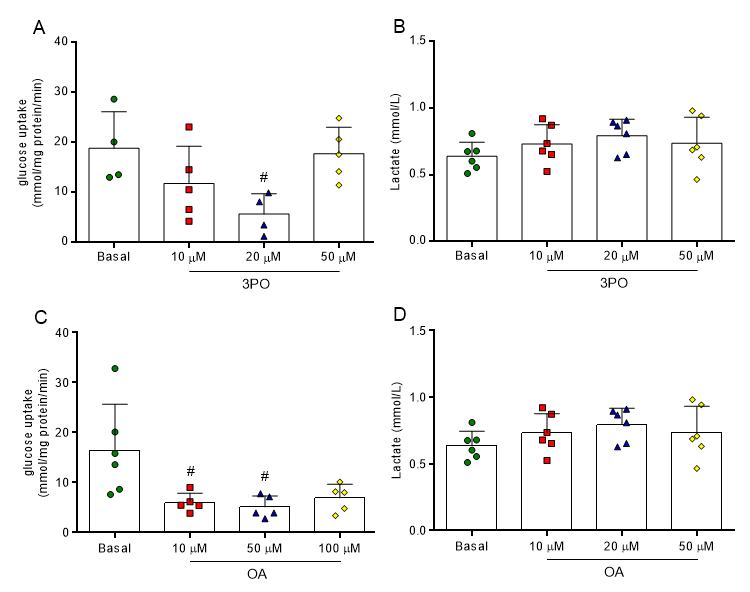

Supplement: Supplementary file 1 — Additional file 1: Figure S1. Dose curve of the effects of glycolytic pathway inhibitors on glucose uptake. Glucose uptake was measured by a radioactivity assay. Lactate medium levels were evaluated by a spectrophotometric method. Dose of 20 μM of 3PO reduces glucose uptake (A), without changing extracellular lactate levels (B). Doses of 10 and 50 μM of oxamic acid (OA) decrease glucose uptake (C), without changing extracellular lactate levels (D). Values are expressed as means ± standard error. Data were analyzed by ANOVA, followed by the Tukey test, assuming P < 0.05. # means significant decrease, when compared to sham group. [file 12974_2022_2612_MOESM1_ESM.tiff]

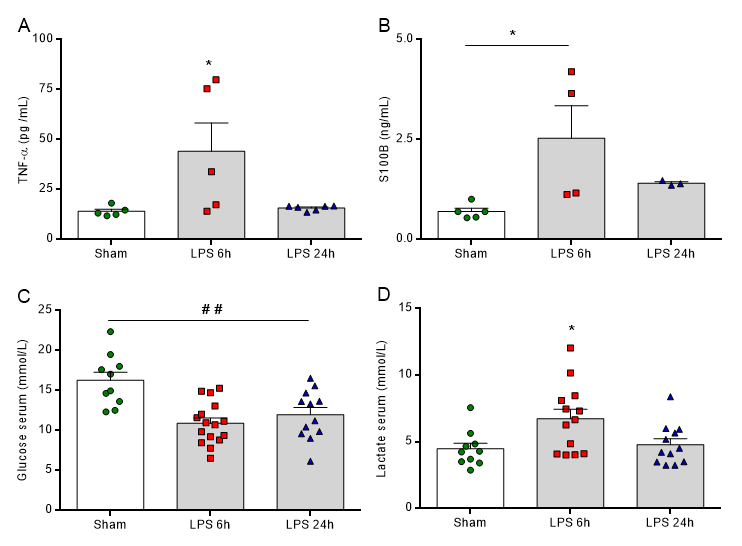

Supplement: Supplementary file 2 — Additional file 2: Figure S2. LPS activates peripheral inflammation. Serum immunocontents of TNF-α and S100B were measured by ELISA. Glucose and lactate serum levels were evaluated by spectrophotometric method. TNF-α (A) and S100B (B) immunocontent serum levels are increased only at 6 h after LPS. Serum glucose levels decreased at both time points (C). Serum lactate levels increased only at 6 h Values are expressed as means ± standard error. Data were analyzed by ANOVA, followed by the Tukey test, assuming P < 0.05. * means significant increase, when compared to sham group (* P < 0.05, ** P < 0.01), # means significant decrease, when compared to sham group. [file 12974_2022_2612_MOESM2_ESM.tiff]

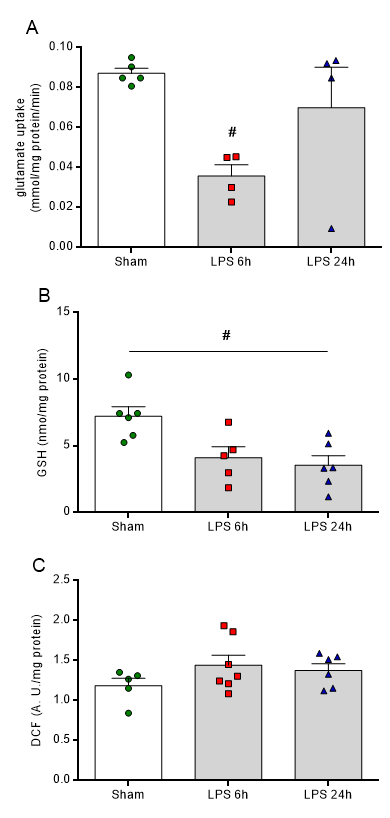

Supplement: Supplementary file 3 — Additional file 3: Figure S3. LPS induces glutamatergic neurotoxicity in the hippocampus. Glutamate uptake was measured by a radioactivity assay. GSH content and total reactive oxygen species (DCF) were measured by a fluorescent method. Administration of LPS decreases glutamate uptake only at 6 h (A). Both time points of neuroinflammation induction reduce non-enzymatic antioxidant defense, GSH content (B), without changes in DCF production (C). Values are expressed as means ± standard error. Data were analyzed by ANOVA, followed by the Tukey test, assuming P < 0.05. # means significant decrease, when compared to sham group. [file 12974_2022_2612_MOESM3_ESM.tiff]

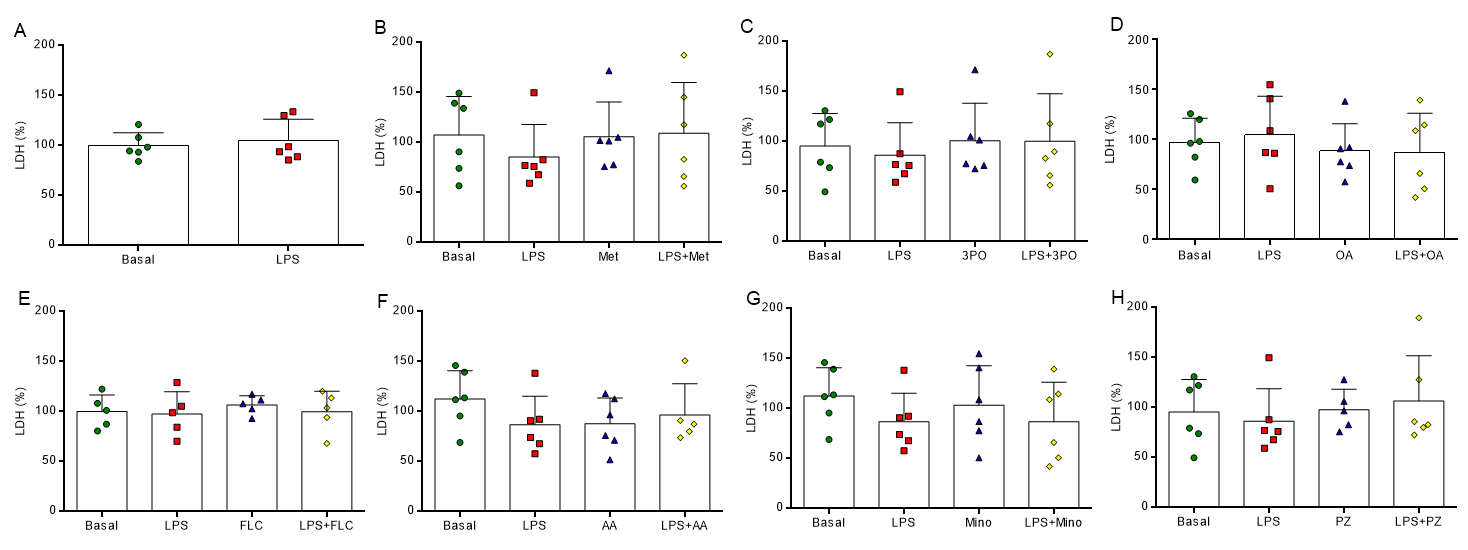

Supplement: Supplementary file 4 — Additional file 4: Figure S4. LPS promotes glutamatergic neurotoxicity in acute hippocampal slices. Glutamate uptake was measured by a radioactivity assay. Protein expressions of glutamate receptor and glutamate transporters (GLAST, GLT1) were analyzed by Western blot. GSH content and total reactive oxygen species (DCF) were measured by a fluorescent method. LPS decreases glutamate uptake (A), regardless of the expressions of the GLAST and GLT1 transporters (B). Neuroinflammation increases NMDAR1 expression (B) and oxidative stress by reducing the non-enzymatic antioxidant defense, GSH content (C), and increases DCF production (D). Values are expressed as means ± standard error. Data were analyzed by Student's unpaired t test, assuming P < 0.05. * means significant increase, when compared to sham group, # means significant decrease, when compared to sham group (# P < 0.05, ## P < 0.01). [file 12974_2022_2612_MOESM4_ESM.tiff]

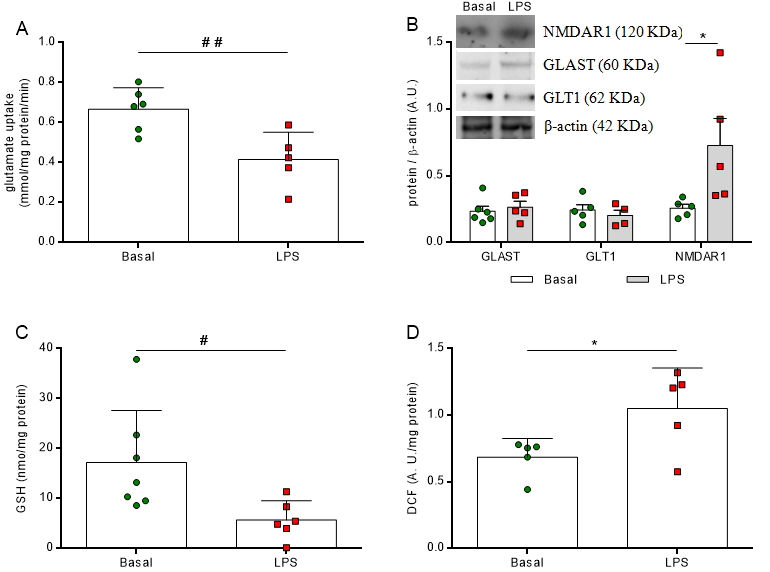

Supplement: Supplementary file 5 — Additional file 5: Figure S5. LPS and inhibitors of energetic metabolism and neuroinflammation do not change cellular integrity in hippocampal slices. Extracellular LDH activity was evaluated by a spectrophotometric method. Incubation of hippocampal slices with the following for one hour in the presence of LPS (10 μg/mL); (A), Metformin (Met, 500 μM) (B), 3PO (20 μM) (C), oxamic acid (OA, 10 μM) (D), fluorocitrate (FLC, 10 μM) (E), arundic acid (AA, 100 μM) (F), minocycline (Mino,10 μM) (G), MCC950 (MCC, 10 μM) (H), or co-incubation, did not alter cellular integrity. Values are expressed as means ± standard error. Data were analyzed by ANOVA, followed by the Tukey test, assuming P < 0.05. [file 12974_2022_2612_MOESM5_ESM.tiff]

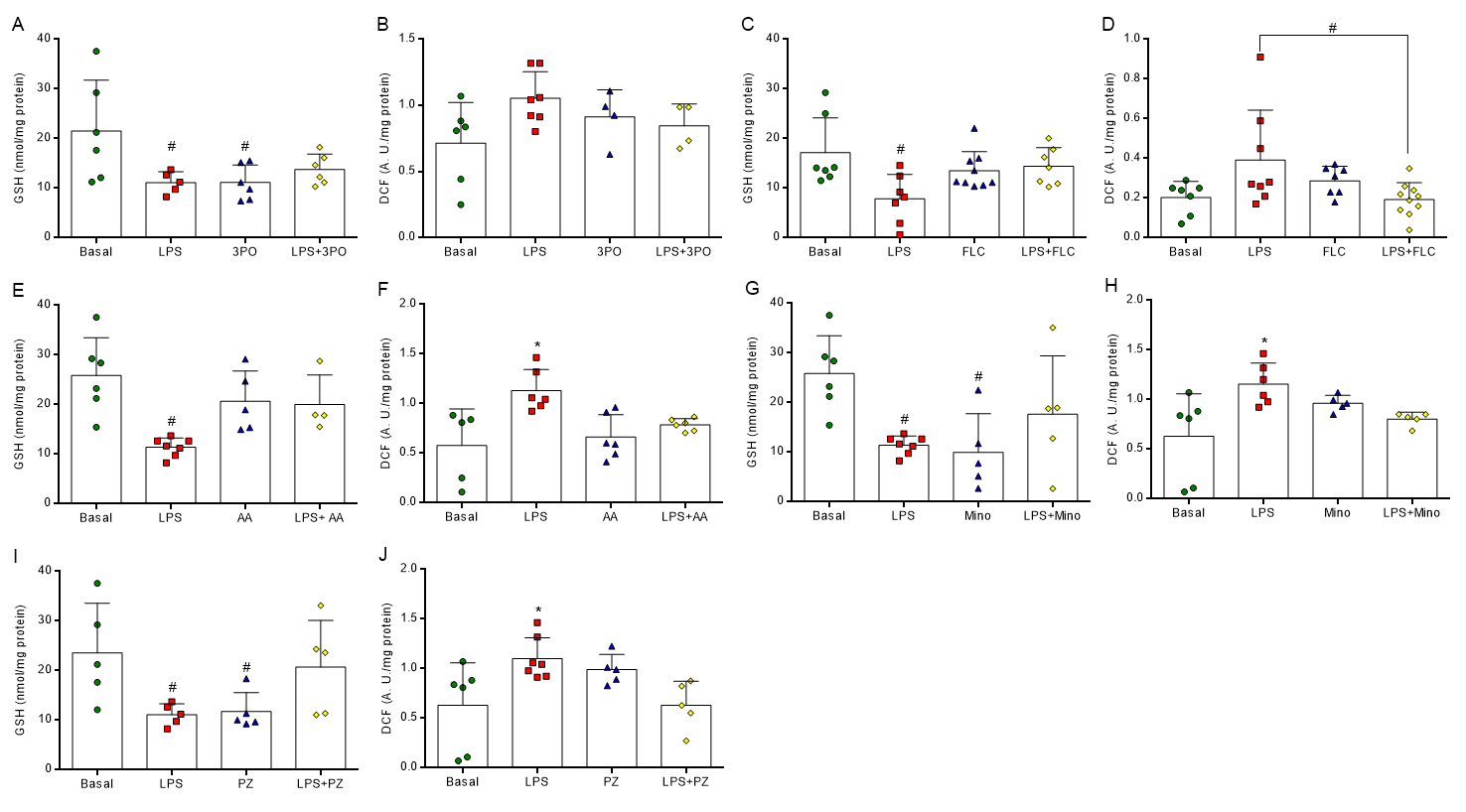

Supplement: Supplementary file 6 — Additional file 6: Figure S6. Effects of metabolic and neuroinflammation inhibitors on LPS-induced oxidative stress in acute hippocampal slices. GSH content and total reactive oxygen species (DCF) were measured by a fluorescent method. The inhibitors 3PO (20 μM) (A-B), fluorocitrate (FLC, 10 μM) (C-D), arundic acid (AA, 100 μM) (E–F), minocycline (Mino,10 μM) (G-H), and MCC950 (MCC, 10 μM)(I-J) reversed the reduction in GSH content and elevation in total reactive oxygen species (DCF) promoted by LPS. Values are expressed as means ± standard error. Data were analyzed by Student's unpaired t test, assuming P < 0.05. * means significant increase, when compared to sham group, # means significant decrease, when compared to sham group (# P < 0.05, ## P < 0.01). [file 12974_2022_2612_MOESM6_ESM.tiff]
