# Supplementary material for: Early effects of LPS-induced neuroinflammation on the rat hippocampal glycolytic pathway
Source: J Neuroinflammation. 2022 Oct 11;19:255. doi: 10.1186/s12974-022-02612-w (PMC9552490; doi:10.1186/s12974-022-02612-w)
Supplement: Supplementary file 7 — Additional file 7: Table S1. List of antibodies used in this study. [file 12974_2022_2612_MOESM7_ESM.docx]

Table 1: List of antibodies used in this study

| Name | Company | Species | Cat. number | RRID | Dilution |
| --- | --- | --- | --- | --- | --- |
| Akt | Cell Signalling | Rabbit, mAb | 4685 | AB_225340 | 1:1,000 |
| Phospho-Akt (Ser473) | Cell Signalling | Rabbit, mAb | 4060 | AB_2315049 | 1:1,000 |
| COX2 | Santa Cruz Biotechnology | Goat, pAb | sc-1745 | AB_631309 | 1:1,000 |
| EAAT2 (GLT1) | Abcam | Rabbit, pAb | ab41621 | [AB_941782](http://antibodyregistry.org/AB_941782) | 1: 5,000 |
| EAAT1 (GLAST) | Abcam | Rabbit, pAb | ab416 | [AB_304334](http://antibodyregistry.org/AB_304334) | 1: 5,000 |
| GFAP | Dako | Rabbit, pAb | Z9334 | AB_2811722 | 1:2000 |
| GLUT1 | Santa Cruz Biotechnology | Goat, pAb | sc-1603 | [AB_2254952](http://antibodyregistry.org/AB_2254952) | 1:2,000 |
| Goat-IgG-HRP | Sigma | Rabbit, unknown | A5420 | [AB_258242](http://antibodyregistry.org/AB_258242) | 1:10,000 |
| HRP-conjugated Actin | Proteintech | Mouse, mAb | HRP60008 | AB_2819183 | 1:20,000 |
| Iba1 | Millipore | Mouse, mAb | MABN92 | [AB_10917271](http://antibodyregistry.org/AB_10917271) | 1:1,000 |
| Mouse-IgG-HRP | GE Healthcare | Goat, unknow | NA931V | [AB_772210](http://antibodyregistry.org/AB_772210) | 1:10,000 |
| p38 MAPK | Sigma | Rabbit, unknown | M0800 | AB_260458 | 1:10,000 |
| Phospho-p38MAPK (Thr18/Tyr182) | Cell Signalling | Rabbit, unknown | 9211 | AB_331641 | 1:10,000 |
| Rabbit-IgG-HRP | GE Healthcare | Monkey, unknow | NA934 | [AB_772206](http://antibodyregistry.org/AB_772206) | 1:10,000 |
| RAGE | Santa Cruz Biotechnology | Goat, pAb | sc-8230 | [AB_2224484](http://antibodyregistry.org/AB_2224484) | 1:1,000 |
| S100B | Sigma | Mouse, mAb | S2532 | [AB_477499](http://antibodyregistry.org/AB_477499) | 1:1000 |
| S100B | Dako | Rabbit, pAb | Z0311 | [AB_10013383](http://antibodyregistry.org/AB_10013383) | 1:5000 |
|  |  |  |  |  |  |
| TLR4 | Santa Cruz Biotechnology | Goat, pAb | sc-16240 | [AB_2205143](http://antibodyregistry.org/AB_2205143) | 1:1,000 |

mAb: monoclonal antibody; pAb: polyclonal antibody
